# A Novel Role of the L-Type Calcium Channel α_1D_ Subunit as a Gatekeeper for Intracellular Zinc Signaling: Zinc Wave

**DOI:** 10.1371/journal.pone.0039654

**Published:** 2012-06-22

**Authors:** Satoru Yamasaki, Aiko Hasegawa, Shintaro Hojyo, Wakana Ohashi, Toshiyuki Fukada, Keigo Nishida, Toshio Hirano

**Affiliations:** 1 Laboratory for Cytokine Signaling, RIKEN Research Center for Allergy and Immunology (RCAI), Yokohama, Kanagawa, Japan; 2 Laboratory of Immune System, Cooperation Program, Graduate School of Frontier Biosciences, Osaka University, Suita, Osaka, Japan; 3 Department of Allergy and Immunology, Graduate School of Medicine, Osaka University, Suita, Osaka, Japan; 4 JST-CREST Program of the Japan Science and Technology Agency, Osaka University, Suita, Osaka, Japan; Cornell University, United States of America

## Abstract

Recent studies have shown that zinc ion (Zn) can behave as an intracellular signaling molecule. We previously demonstrated that mast cells stimulated through the high-affinity IgE receptor (FcεRI) rapidly release intracellular Zn from the endoplasmic reticulum (ER), and we named this phenomenon the “Zn wave”. However, the molecules responsible for releasing Zn and the roles of the Zn wave were elusive. Here we identified the pore-forming α_1_ subunit of the Cav1.3 (α_1D_) L-type calcium channel (LTCC) as the gatekeeper for the Zn wave. LTCC antagonists inhibited the Zn wave, and an agonist was sufficient to induce it. Notably, α_1D_ was mainly localized to the ER rather than the plasma membrane in mast cells, and the Zn wave was impaired by α_1D_ knockdown. We further found that the LTCC-mediated Zn wave positively controlled cytokine gene induction by enhancing the DNA-binding activity of NF- κB. Consistent with this finding, LTCC antagonists inhibited the cytokine-mediated delayed-type allergic reaction in mice without affecting the immediate-type allergic reaction. These findings indicated that the LTCC α_1D_ subunit located on the ER membrane has a novel function as a gatekeeper for the Zn wave, which is involved in regulating NF-κB signaling and the delayed-type allergic reaction.

## Introduction

Zn is an essential trace element. Approximately 10% of all the genes in the human genome may contain Zn-binding motifs [1], and the dysregulation of Zn homeostasis is linked to a wide range of physiological defects, including those affecting growth, development, and the immune system [2], [3].

Recent advances have revealed the existence and importance of free or labile Zn in living organisms [4], and Zn has been increasingly recognized as a potential biological signaling molecule [5]. It is well established that synaptic Zn acts as a neurotransmitter that can mediate cell–to-cell communication [6], [7], [8]. In addition to such intercellular communication, Zn can act as a second messenger [9], capable of transducing extracellular stimuli into intracellular signaling events. Intracellular Zn signaling is classified into two types: early and late [5], [10], [11]. Late Zn signaling, which occurs several hours after extracellular stimulation, depends on changes in the expression profile of Zn-related molecules, such as Zn transporters and metallothioneins, and leads to alterations in the intracellular Zn content and/or intracellular distribution of Zn [12], [13], [14], [15], [16]. On the other hand, early Zn signaling occurs several minutes after extracellular stimulation and does not involve transcriptional changes. It is mediated by extracellular Zn’s influx into the cytoplasm and by intracellular Zn’s detachment from metalloproteins and release from intracellular organelles.

FcεRI stimulation induces a rapid elevation of the intracellular free Zn level in mast cells, and we named this phenomenon the “Zn wave” [9]. The Zn wave originates in the perinuclear region, which includes the endoplasmic reticulum (ER). Our evidence suggests that it is positively involved in FcεRI-mediated cytokine production in mast cells. These findings indicated a novel function for the Zn released from intracellular organelles as an intracellular second messenger, like Ca^2+^ [9]. However, the gatekeeper for the Zn wave remained unknown.

In addition to the FcεRI-mediated Zn wave in mast cells, the rapid elevation of intracellular Zn by several stimuli for certain cellular functions has been reported [17], [18], [19]. However, the mechanism for the rapid intracellular induction of free Zn in those studies, as well as in the case of the Zn wave, has remained unclear.

L-type calcium channels (LTCCs) can conduct Zn [20] and act as Zn-permeable channels on the plasma membrane of neurons and pancreatic β cells [21], [22]. However, it is unclear whether LTCCs can also function in Zn’s release from intracellular organs. The LTCCs are complexes that include α_1_, β, and α_2_/δ subunits. The α_1_ subunit functions as the voltage sensor, selective filter, and ion-conducting pore [23], and α_1_ subunit on the cell surface is proposed to require an association with the β subunit, which masks one or more ER-retention signals [24], [25]. Taken together, these characteristics of LTCCs make them potential candidates for performing the Zn wave gatekeeper function [21], [22].

Transcription factors of the nuclear factor κB (NF-κB)/Rel family play pivotal roles in inflammatory and immune responses [26], [27]. In unstimulated cells, NF-κB is sequestered in the cytoplasm by its inhibitory proteins, the IκBs. Stimulants that activate the NF-κB pathway induce the phosphorylation and degradation of IκBs through the ubiquitin-proteasome pathway, releasing NF-κB to enter the nucleus, where it binds specific DNA sequences [28]. Mast cells secrete cytokines in response to antigen stimulation and other activators [29], [30]. NF-κB acts as a key regulator for inflammatory cytokines such as IL-6 and TNF-α [31]; in mast cells, FcεRI stimulation induces the nuclear translocation of NF-κB to increase these cytokines [32].

Redox regulation of NF-κB’s DNA-binding activity by Zn has also been demonstrated; the mechanism involves Zn’s binding to cysteine residues in the DNA-binding region of NF-κB, as shown by site-directed mutagenesis experiments [33], [34], [35]. These findings suggest that Zn is a signaling molecule that modulates NF-κB, although the link between the regulation of NF-κB activation and the Zn wave is unknown.

Here, we show that the pore-forming α_1D_ subunit of LTCC on the ER membrane plays a novel role in generating the Zn wave, and that the NF-κB signaling pathway, a key regulator of allergic responses, is one of the targets of the LTCC–mediated Zn wave in mast cells.

## Results

### Expression of the LTCC α_1D_ Subunit on the ER Membrane in Mast Cells

**Figure 1:**
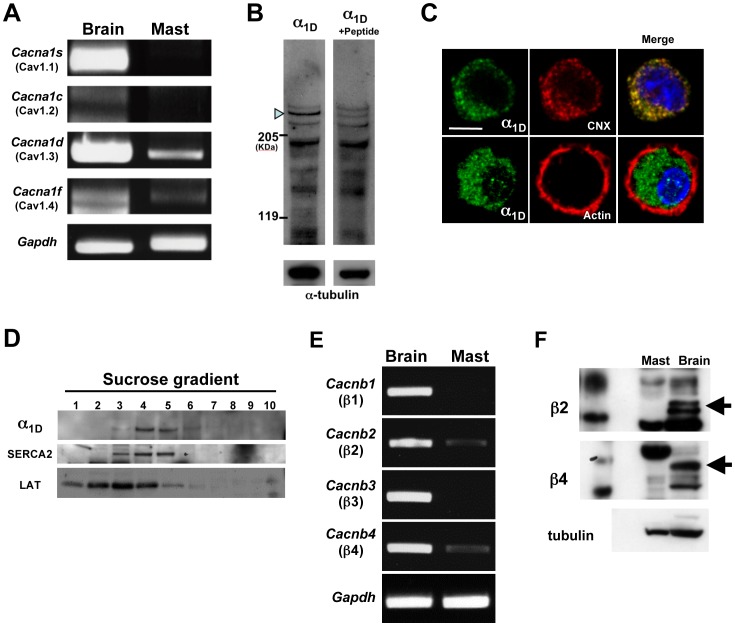
The α_1D_ subunit of LTCC is primarily expressed on the ER membrane in mast cells. (A) RT-PCR of mRNA encoding the α_1_ subunit of LTCC family members (*cacna1s, cacna1c, cacna1d, and cacna1f*), and *Gapdh* in BMMCs. (B) Western blot for α_1D_ in BMMCs. Total cell lysates were blotted with an anti-α_1D_ polyclonal antibody or the same antibody pre-incubated with an antigenic peptide. Arrowhead indicates the putative α_1D_ signal; this signal detected by the antibody pre-incubated with antigenic peptide was 15.1±11.1% of that detected by the anti-α_1D_ polyclonal antibody. (C) Intracellular distribution of the LTCC α_1D_ subunit in BMMCs examined by confocal microscopy. Representative images are shown. Staining with an anti-α_1D_ monoclonal antibody is in green, anti-Calnexin (ER marker) in red, or phalloidin-Alexa 546 (for F-actin beneath the plasma membrane) in red, and 4′ 6-diamidino-2-phenylindole, dihydrochloride (DAPI; for nuclei) in blue. (D) The postnuclear supernatant obtained from BMMCs was fractionated by ultracentrifugation in a discontinuous sucrose gradient. The collected fractions were then separated by SDS-PAGE, and the protein distributions were detected by immunoblotting with antibodies against α_1D_, SERCA2 (ER), and LAT (plasma membrane). (E) The mRNA expression of LTCC β-subunit family members (*cacnb*1 to *cacnb*4) in the brain and BMMCs was examined by RT-PCR. (F) The protein levels of the β2 and β4 subunits in the brain and BMMCs were examined by western blots.

LTCCs are Zn-permeable [20] and are expressed by various cell types, including non-excitable cells [36]. To determine whether this ion channel could be involved in the Zn wave in mast cells, we examined the expression of the four pore-forming α_1_ subunits. RT-PCR analysis showed that *cacna1d,* the α_1_ subunit for the Cav1.3 LTCC (α_1D_), was the dominantly expressed α_1_ subunit in bone marrow-derived mast cells (BMMCs) (Figure 1A). Furthermore, α_1D_ protein was observed in BMMCs by western blot analysis with an antibody against α_1D_; the signal was abolished by prior incubation of the antibody with an antigenic peptide (Figure 1B).

We next examined the intracellular distribution of the α_1D_ in BMMCs. In confocal microscopic analysis, the immunofluorescent signal of α_1D_ was observed in the intracellular area, partially merged with that of the ER marker calnexin, but not with that of F-actin, which accumulated beneath the plasma membrane (Figure 1C). In addition, in a discontinuous sucrose density gradient ultracentrifugation experiment, α_1D_ was distributed in fractions 4 and 5, which corresponded with fractions containing the ER marker SERCA2 (fractions 3 to 5), but only partially overlapped with fractions containing the plasma membrane marker LAT (fractions 1 to 4) (Figure 1D). These results indicated that α_1D_ localized preferentially to intracellular organelle membranes, such as the ER membrane, rather than to the plasma membrane in mast cells.

The β subunit is known to be required for α_1_ subunits to be localized to the plasma membrane and for their full activity as a channel [24], [25]. As shown in Figures 1E and F, the expression levels of β subunits were very low in BMMCs, consistent with α_1D_’s main localization to the ER in these cells. This intracellular localization of α_1D_ in BMMCs suggested that it plays a different role from that observed in other cells, in which it is located on the plasma membrane and acts as a calcium channel.

### The LTCC α_1D_ Subunit is a Gatekeeper for the Zn Wave in Mast Cells

**Figure 2:**
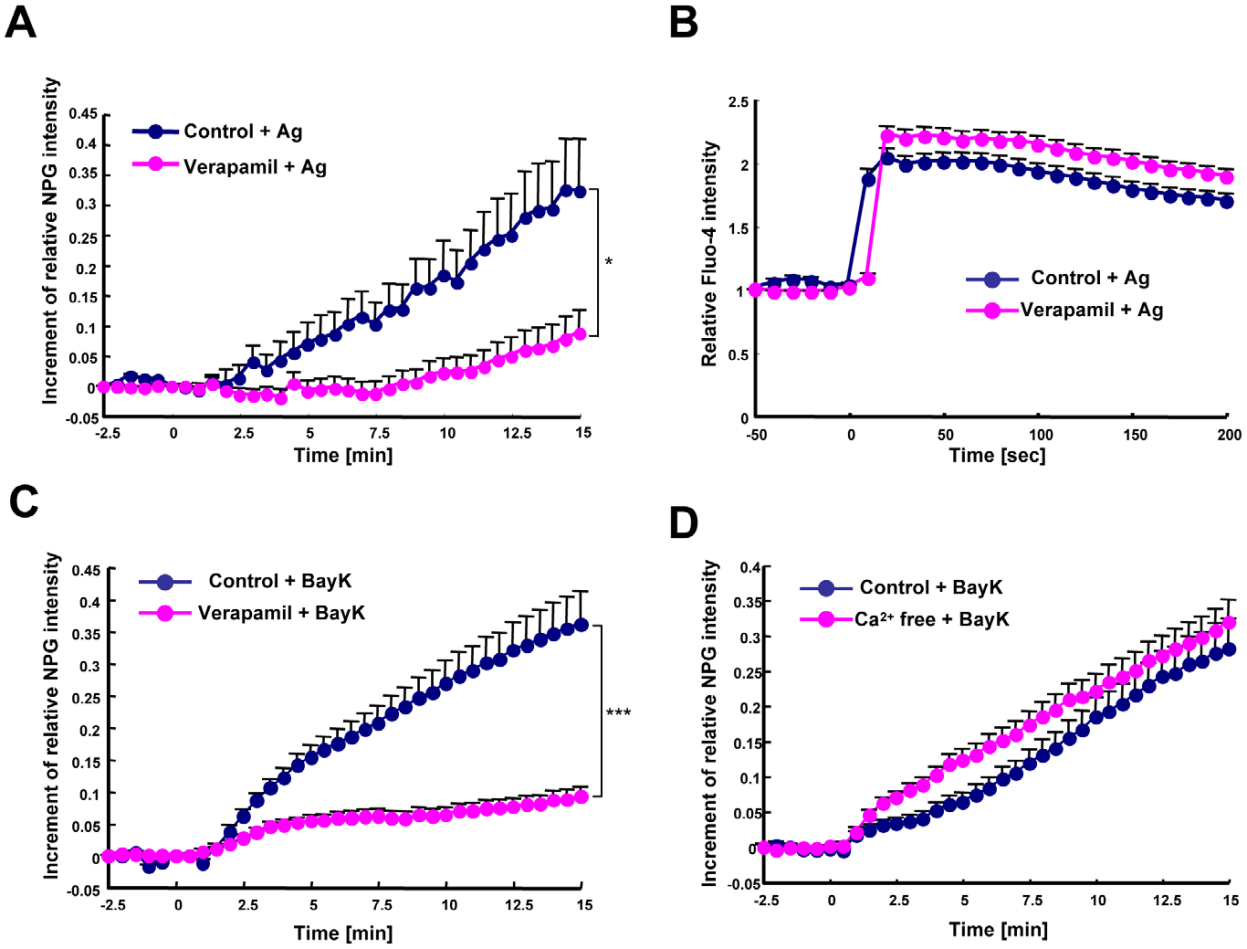
LTCC is involved in regulation of the Zn wave. (A) The intracellular labile Zn level after FcεRI-mediated stimulation was examined using the fluorescent Zn indicator Newport Green in mast cells with or without pre-treatment with 100 µM Verapamil, an LTCC antagonist. The data represent the relative fluorescent intensity of Newport Green. The difference in Newport Green intensity at 15 min between the control and Verapamil-treated BMMCs was statistically significant. *P<0.05, Student’s *t*-test. (B) The FcεRI-mediated Ca^2+^ elevation in control and Verapamil-treated BMMCs was examined using the fluorescent Ca^2+^ indicator Fluo-4. Data represent the relative fluorescent intensity of Fluo-4. The difference in Fluo-4 intensity between the control and Verapamil-treated BMMCs was not statistically significant. (C) The intracellular labile Zn level upon treatment with the LTCC agonist (s)-(-)-BayK8644 in BMMCs with or without 100 µM Verapamil. The difference in Newport Green intensity at 15 min between the control and Verapamil-treated BMMCs was statistically significant. ***P<0.001. (D) The intracellular labile Zn level upon (s)-(-)-BayK8644 treatment of BMMCs in control or Ca^2+^-free Tyrode’s buffer. The difference in Fluo-4 intensity between the control and Ca^2+^-free Tyrode’s buffer samples was not statistically significant. All data are representative of at least three experiments, and are shown as the mean + SEM. NPG, Newport Green; Vera, Verapamil; Bay, (s)-(-)-BayK8644.

To examine whether LTCCs expressed on the ER membrane are involved in the Zn wave, we examined the effect of the LTCC antagonist Verapamil on the FcεRI-induced Zn wave in BMMCs. Verapamil-treated BMMCs showed an impaired Zn wave compared to control cells (Figure 2A), without disturbing cell survival or FcεRI expression (Figures S1A and B). The FcεRI-mediated Ca^2+^ elevation, however, was not inhibited by Verapamil in BMMCs (Figure 2B). The Zn wave was also inhibited in BMMCs treated with a lower concentration of Verapamil (1 µM) or with another type of LTCC antagonist, Diltiazem (Figures S2A and B), and Diltiazem did not affect the FcεRI-mediated Ca^2+^ elevation (Figure S2C). On the other hand, treatment with the LTCC agonist (s)-(-)-BayK8644, without antigen stimulation, induced an elevation in the intracellular Zn level, but not in the Ca^2+^ level (Figures 2C, D, S3A, and B). The LTCC agonist-induced increase in intracellular Zn was observed even in the absence of Ca^2+^, and it was inhibited by Verapamil (Figures 2C, D, and S3C). To reveal whether the FcεRI-induced Zn wave and LTCC-mediated Zn elevation were regulated by a similar mechanism, BMMCs were stimulated with antigen in the presence of the LTCC agonist. The level of the FcεRI-induced Zn elevation was similar in BMMCs with or without the LTCC agonist, indicating that the FcεRI-induced Zn wave and LTCC agonist-induced Zn elevation probably occur by a similar mechanism (Figure S3D).

**Figure 3:**
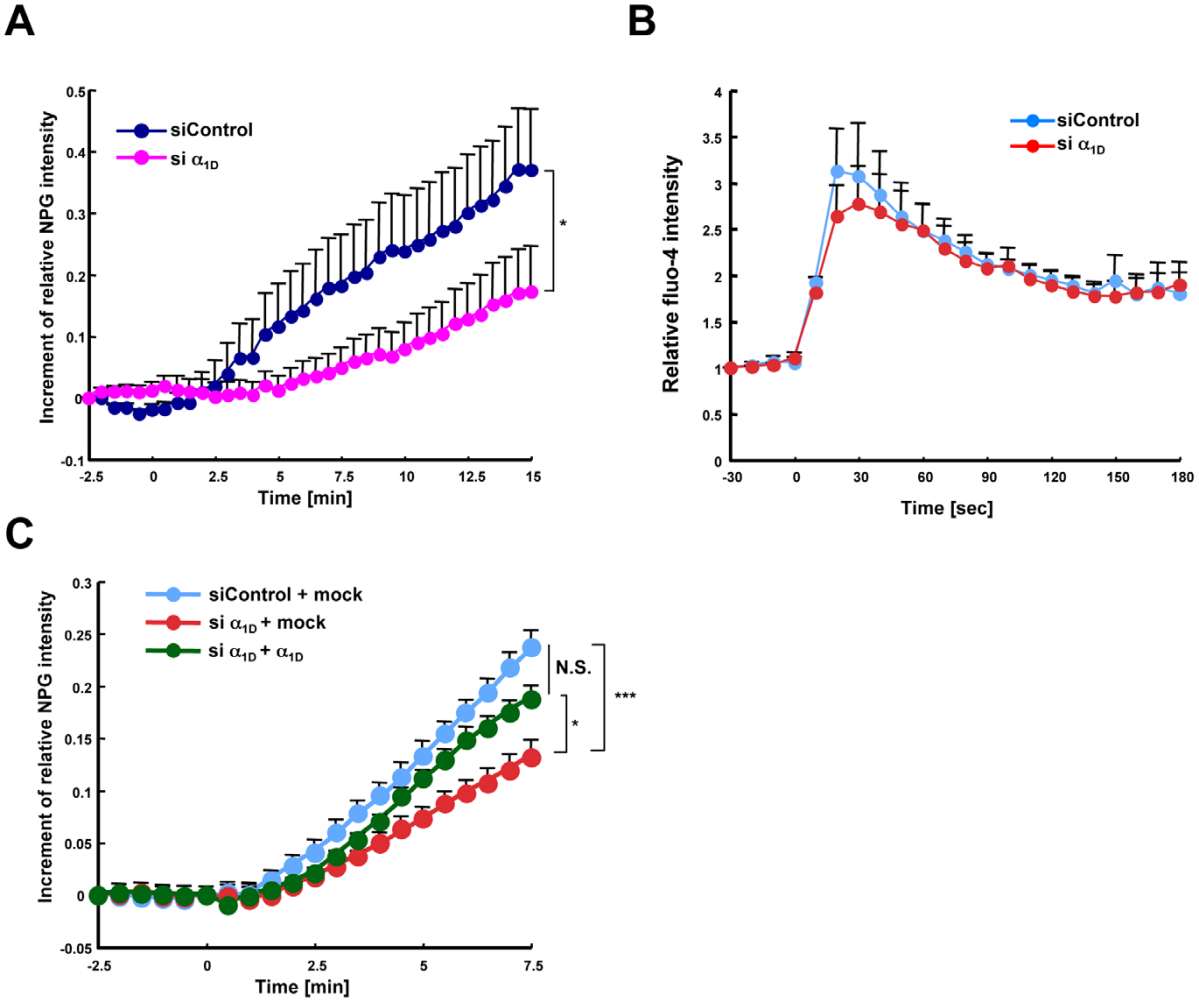
Effect of α_1D_ knockdown on the FcεRI-mediated Zn wave. (A) The intracellular labile Zn level upon antigen stimulation was examined in control and α_1D_ siRNA-treated BMMCs. The difference in Newport Green intensity at 15 min between the control and α_1D_ siRNA-treated BMMCs was statistically significant. *P<0.05, Student’s *t*-test. (B) The intracellular Ca^2+^ level upon antigen stimulation was examined in control and α_1D_ siRNA-treated BMMCs. The difference in Fluo-4 intensity between the control and α_1D_ siRNA-treated BMMCs was not statistically significant. (C) The FcεRI-mediated Zn wave was examined in α_1D_ siRNA-treated BMMCs with or without transfection of human α_1D_. All data represent the mean + SEM. N.S., not significant, *P<0.05, ***P<0.001, Bonferroni’s multiple comparison test. NPG, Newport Green.

All these results were consistent with the idea that α_1D_, one of the α_1_ subunits of LTCCs, might be a gatekeeper for the Zn wave in mast cells. To examine this possibility further, we knocked down α_1D_ in BMMCs by siRNA. The expression level of the mRNA for *Cacna1d*, but not for other *Cacna1* family members, such as *Cacna1f*, and the protein level of α1_D_ were reduced in the α_1D_-knockdown BMMCs compared with control cells (Figures S4). The FcεRI-induced Zn wave was significantly reduced in the α_1D_-knockdown BMMCs compared with control cells (Figure 3A). On the other hand, the α_1D_ knockdown did not affect the FcεRI-induced Ca^2+^ elevation (Figure 3B), similar to the results of Verapamil treatment. Moreover, the ectopic expression of wild-type α_1D_ rescued the inhibitory effect of siRNA knockdown on the Zn wave (Figure 3C). These results indicated that the LTCC α_1D_ subunit is a gatekeeper for the Zn wave.

### Requirement of the Zn Wave for FcεRI-induced Cytokine Gene Induction, but not for Degranulation in Mast Cells

**Figure 4:**
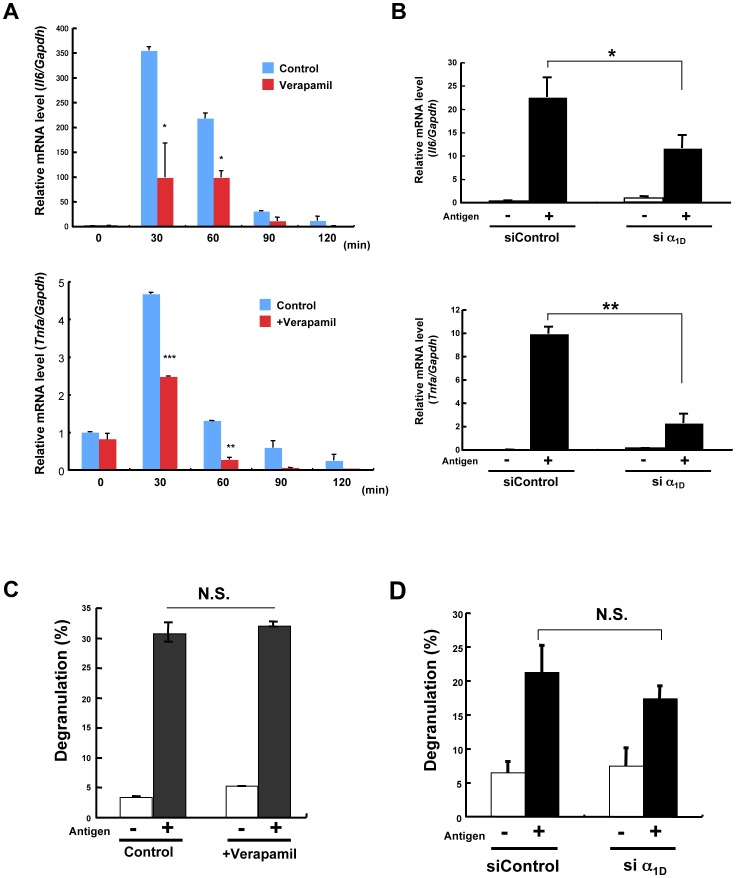
The LTCC-mediated Zn wave is involved in cytokine gene inductions. (A) The FcεRI-mediated inductions of *Il6* and *Tnfa* transcription upon antigen stimulation for the indicated time in BMMCs with or without pretreatment with 100 µM Verapamil were determined by semi-quantitative RT-PCR. (B) The mRNA levels of *Il6* and *Tnfa* upon antigen stimulation for 60 min in control (siControl) or α_1D_ siRNA-treated (si α_1D_) BMMCs were determined by semi-quantitative RT-PCR. (C) The level of degranulation after 30 min of antigen stimulation in BMMCs with or without pretreatment with 100 µM Verapamil was determined by measuring the β-hexosaminidase activity. (D) Level of degranulation after 30 min of antigen stimulation in siControl and α1_D_ siRNA-treated BMMCs. N.S., not significant, *P<0.05, **P<0.01, two-tailed Student’s *t*-test.

FcεRI stimulation activates several downstream pathways that initiate immediate allergic inflammatory responses by eliciting mast-cell degranulation, accompanied by the rapid release of preformed chemical mediators, such as histamine and serotonin. In contrast, the mast cell–mediated delayed-type responses are mainly dependent on cytokine production. To examine whether the LTCC-mediated Zn wave could play a role in these mast-cell-activation events, we first investigated the effect of Verapamil treatment and α_1D_ knockdown on FcεRI-mediated cytokine gene induction and degranulation. Inhibition of the Zn wave by Verapamil reduced the FcεRI-mediated gene induction of *Il6* and *Tnfa* in BMMCs (Figure 4A). The α_1D_-knockdown BMMCs also showed impaired FcεRI-mediated gene induction of *Il6* and *Tnfa* (Figure 4B). In these siRNA experiments, the *Cacna1d* mRNA expression level in the α_1D_-knockdown BMMCs was 20.5±7.1% of the control level. On the other hand, neither Verapamil treatment nor α_1D_ knockdown inhibited the FcεRI-mediated degranulation in these cells (Figures 4C and D). Taken together, these results indicated that the Zn wave is involved in the FcεRI-mediated cytokine gene induction, but not the degranulation, of mast cells.

### Role of the Zn Wave in the NF-κB Pathway

**Figure 5:**
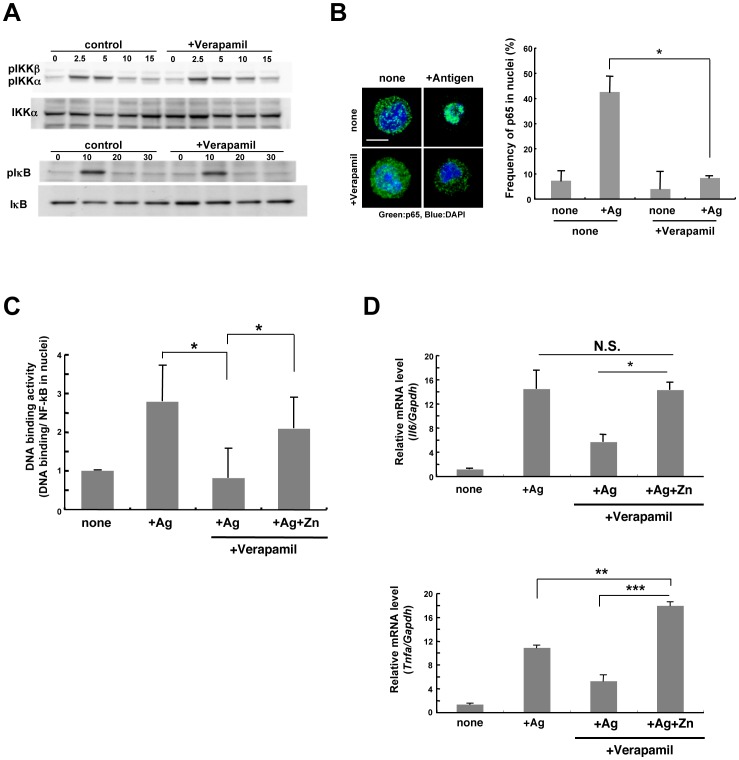
Possible involvement of the Zn wave in regulating the DNA-binding activity of p65 NF-κB. (A) IKKα/β phosphorylation and IκBα phosphorylation and degradation. After antigen stimulation, cells were lysed, and the cytosol fraction was immunoblotted with anti-phospho-IκBα or anti-phospho-IKKα/β antibodies. Results were normalized to the non-phosphorylated IκBα and IKKα. (B) NF-κB nuclear translocation visualized by confocal microscopy. BMMCs were untreated or treated with Verapamil and stimulated with antigen for 15 min. Cells were stained with anti-p65 (green) and DAPI (blue). Scale bar, 10 µm. The frequency of NF-κB nuclear-translocated cells was calculated. Values are means + S.D. *P<0.05, two-tailed Student’s *t*-test. (C) DNA-binding activity was calculated by dividing the amount of p65 bound to target DNA by the total p65 in the nuclear fraction. BMMCs were untreated or pre-treated with Verapamil, then stimulated with antigen for 15 min. For Zn supplementation, 1 µM Zn and pyrithione were added with the antigen stimulation. (D) BMMCs were untreated or treated with Verapamil, and stimulated with antigen for 60 min. Zn supplementation was as in (C). The *Il6* and *Tnfa* mRNA levels were determined by semi-quantitative RT-PCR. Data show means + S.D. N.S., not significant, *P<0.05, **P<0.01, ***P<0.001, Bonferroni’s multiple comparison test.

Since NF-κB is a master transcription factor that controls the expression of proinflammatory cytokines such as IL-6 and TNF-α in mast cells [37], the Zn wave was likely to be involved in the FcεRI-induced NF-κB-signaling pathway. Inhibiting the Zn wave with Verapamil did not affect FcεRI-induced IKK phosphorylation, IκB phosphorylation, or its degradation in BMMCs (Figure 5A). Even though the upstream activation pathway of NF-κB was intact, the frequency of NF-κB p65 accumulation in the nuclei upon FcεRI stimulation was reduced in the Verapamil-treated BMMCs (Figure 5B). These results indicated that the Zn wave might be required for NF-κB’s localization to nuclei, but not for its upstream activation pathway. We further investigated whether the Zn wave was required for the nuclear import step. For this, we used the exportin inhibitor leptomycin B (LMB). The frequency of NF-κB in nuclei was elevated by LMB treatment, and this effect was observed even in the presence of Verapamil (Figure S5A), suggesting that the Zn wave might be required for post nuclear translocation events, rather than for the nuclear import step.

Therefore, we next examined the DNA-binding activity of NF-κB p65 in nuclei. Whereas the DNA-binding activity of NF-κB in nuclei was elevated after FcεRI stimulation in BMMCs, it was reduced in Verapamil-treated cells. This reduction in DNA-binding activity was recovered by adding Zn with the FcεRI stimulation (Figure 5C). Consistent with this result, the reduction in FcεRI-mediated cytokine gene induction in Verapamil-treated BMMCs was recovered by Zn supplementation (Figure 5D). These results indicated that the Zn wave might participate in the signal transduction for cytokine gene induction by enhancing the DNA-binding activity of NF-κB.

### Role of the Zn Wave in the Regulation of Allergic Responses *in vivo*

**Figure 6:**
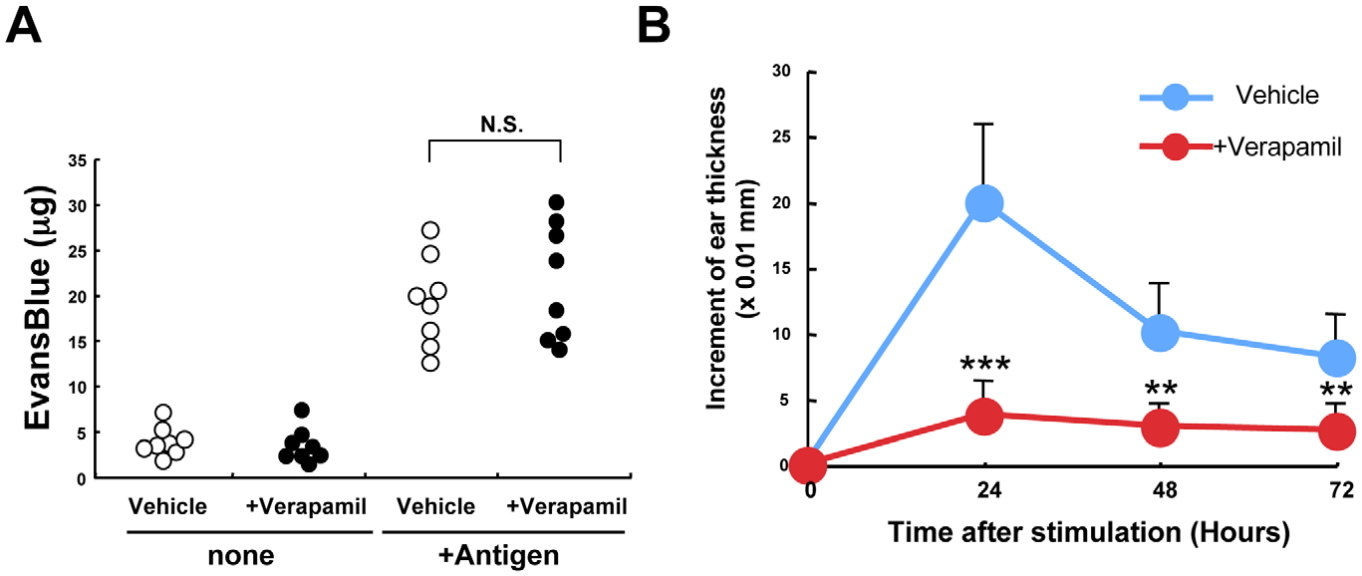
Effect of Verapamil treatment on allergic responses in vivo. (A) Analysis of passive cutaneous anaphylaxis, an immediate-type allergy response. Vehicle- or Verapamil-treated mice received intradermal injections of IgE anti-DNP into the right ear and saline into the left (control). Data show the amount of Evans blue extravasation into the ears. (n = 8 per group, from two independent experiments). N.S., not significant. (B) Analysis of contact hypersensitivity, a delayed-type allergic response. Vehicle- or Verapamil-treated mice were sensitized with FITC, and ear swelling was measured at the indicated times after hapten challenge. Data show means + SEM. (n = 5 per group, experiment performed three times). **P<0.01, ***P<0.001, two-tailed Student’s *t*-test.

Mast cells are a major player in allergic responses such as the immediate- and delayed-type hypersensitivity reactions [30]. Passive cutaneous anaphylaxis (PCA) and contact hypersensitivity (CHS) are mouse models for the immediate-type and delayed-type allergic responses, respectively. We therefore examined the effect of Verapamil treatment on the PCA and CHS reactions. The allergic response in the PCA model was evaluated by the extravasation of Evans blue dye in the ears of mice that had been sensitized and challenged with an antigen. There was no notable difference in the extravasation of Evans blue dye in the ear of the vehicle-versus Verapamil-treated mice (Figure 6A), indicating that the mast cell–mediated PCA reaction occurred normally in the presence of Verapamil.

We then examined the CHS response to the experimental hapten FITC, by assessing the amount of tissue swelling at the site of hapten challenge. While the vehicle-treated mice developed a robust CHS response 24 h after stimulation, the Verapamil-treated mice showed a greatly reduced response (Figure 6B). In addition, mice treated with the other LTCC antagonist, Diltiazem, showed a decreased FITC-induced CHS response (Figure S6). The effects of Verapamil on mast-cell activation *in vitro* (Figure 4) and the allergic response *in vivo* together indicated that the Zn wave plays a role in FcεRI-induced cytokine production, but not in degranulation, and is involved in regulating the delayed-type but not the immediate–type allergic response.

## Discussion

### Identification of the LTCC α_1D_ Subunit as a Gatekeeper for the Zn Wave

It is well established that LTCCs function as voltage-gated calcium channels on the plasma membrane. In this study, we showed that the LTCC α_1D_ subunit was expressed in mast cells, but was localized to the ER rather than to the plasma membrane. We also showed that the expression level of LTCC β subunits, which are required for the localization of α_1_ subunits to the plasma membrane [24], [25], was very low in mast cells. Furthermore, the expression of ZnT-1 in mast cells [16] might support the ER localization of α_1D_ subunits, because ZnT-1 is reported to interact with β subunits on the plasma membrane, reducing their availability to bind α_1_, and thus inhibiting α_1_-subunit trafficking to the plasma membrane [38].

An important finding in the present study was that the α_1D_ subunit expressed on the ER membrane has little effect on FcεRI-induced Ca^2+^ influx, and instead plays a novel role as a gatekeeper for the Zn wave. Our data showed that LTCC antagonist treatment or α_1D_ knockdown inhibited the Zn wave, but did not affect the FcεRI-mediated Ca^2+^ elevation or FcεRI-mediated degranulation, which requires an increase in intracellular Ca^2+^. In addition to the lack of effect on FcεRI-mediated Ca^2+^ elevation, Ca^2+^-mediated signaling was not disturbed in the Verapamil-treated BMMCs, as shown by the normal nuclear translocation of NFAT2 in these cells (Figure S7). In addition, LTCC agonist treatment increased the level of intracellular free Zn but not of Ca^2+^ in mast cells. These results showed that LTCC is not involved in the FcεRI-mediated Ca^2+^ regulation in mast cells. This might be because mast cells, like lymphocytes, utilize store-operated calcium (SOC) entry as their main mode of Ca^2+^ influx [39]. However, we could not rule out the ability of α_1D_ subunit expressed on the ER membrane to conduct Ca^2+^ from the ER to the cytoplasm.

Most importantly, our finding that the LTCC α_1D_ subunit, when expressed on the ER membrane, has a novel function as a gatekeeper for the Zn wave also made it possible for us to address the physiological roles of the Zn wave.

### Regulation of the LTCC Activation for Zn Wave Generation

The α_1_ subunits of LTCC contain a voltage-sensor domain, and the channel activity is elevated after membrane depolarization. The plasma membrane potential in BMMCs is hyperpolarized after FcεRI stimulation [40], [41], but we found that inhibition of the FcεRI-mediated plasma membrane hyperpolarization by high KCl treatment did not impair induction of the Zn wave (Figures S8A and B). We then examined the intracellular membrane potential using tetramethyl rhodamine methyl ester (TMRM). Treatment with the ADP/ATP transporter inhibitor bongkrekic acid inhibited the FcεRI-mediated intracellular membrane depolarization, but it did not inhibit the induction of the Zn wave (Figures S8C and D). This result suggested that intracellular membrane depolarization does not affect the Zn wave generation, although we cannot exclude the possibility that depolarization of the ER inner membrane has an effect.

Modification of the pore-forming α_1_ subunit by phosphorylation has an additional effect on channel activity; in fact, cAMP-mediated channel activity is reduced by site-directed mutagenesis of the PKA consensus sites of α_1D_ [42]. However, we did not observe a negative effect on the Zn wave by PKA inhibitor treatment (Figure S9); therefore, PKA may not participate in the regulation of the Zn wave, at least in mast cells. As-yet unidentified regulatory proteins on the ER membrane may control this event.

### Zn Wave Regulates the DNA-binding Activity of NF-κB and Cytokine Gene Induction

We found that the LTCC-mediated intracellular Zn signal upregulates the DNA-binding activity of NF-κB and the transactivation of inflammatory cytokines. NF-κB-mediated transactivation can be divided into the following three steps. First, NF-κB dissociates from IκB after IκB’s phosphorylation and degradation. Second, NF-κB translocates from the cytosol to the nucleus, and finally, NF-κB binds to its target sequences. We found that the frequency of NF-κB p65 nuclear translocation was reduced in LTCC antagonist-treated cells, even though the upstream regulators were unaffected. Treatment with the exportin inhibitor LMB enhanced the frequency of NF-κB in nuclei, and this effect was observed in LTCC antagonist- and LMB-treated cells, suggesting that the Zn wave is not involved in the nuclear translocation step. Rather, our evidence indicates that the Zn wave is required for the DNA-binding activity of NF-κB.

That the LTCC antagonist treatment reduced the DNA-binding activity of NF-κB further supports this scenario. Moreover, the DNA-binding activity of NF-κB was enhanced by supplementing the cell lysate with Zn (Figure S5B). These findings suggest that the elevated intracellular Zn caused by the Zn wave positively regulates the DNA-binding activity of NF-κB. However, although we showed that the Zn wave is involved in FcεRI-mediated cytokine gene induction, LTCC agonist treatment, which induced an increase in free zinc, like the Zn wave, did not increase the mRNA induction and protein synthesis of IL-6 and TNF-α (Figure S10). These results suggest that the Zn wave is required, but not sufficient, for the FcεRI-induced cytokine productions in mast cells. Nevertheless, taken together, our results indicate that the Zn wave is a novel modulator of NF-κB activation.

### The Zn Wave is Involved in the Delayed-type Allergic Response *in vivo*

The mast cell is one of the effector cells for allergic responses *in vivo*. Mast cell-derived cytokines, which are induced by FcεRI-mediated activation of the PKC/Bcl10/Malt1/NF-κB signaling pathway, are known to be involved in delayed-type allergic responses, such as CHS [16], [43]. Mast cell-derived TNF is required for the maximum CHS response; it induces the infiltration of leukocytes at the site of inflammation [44], enhances the elongation of cutaneous nerves [45], and enhances the dendritic cell migration to draining lymph nodes [46]. In this study, we revealed that treating mice with the LTCC antagonist Verapamil inhibited the CHS reaction, a delayed-type immune response, without affecting the PCA, an immediate-type response. Consistent with these results, we showed that Verapamil treatment inhibited the FcεRI-mediated activation of NF-κB’s DNA-binding activity and cytokine gene inductions, but not Ca^2+^ elevation or degranulation in BMMCs. Thus, the inhibitory effect of Verapamil on CHS might depend at least in part on the reduction of mast cell-derived cytokine production, independent of other mediators, such as histamine. Although we do not exclude the possibility that Verapamil affects the function of dendritic cells and T cells in vivo, all our results suggest that one of the *in vivo* roles of the Zn wave is to regulate the allergic response by controlling cytokine production in mast cells.

In summary, we identified a novel function of the pore-forming α_1D_ subunit of LTCC, when it is expressed on the ER membrane, as the gatekeeper for the Zn wave in mast cells. In addition, the LTCC-mediated Zn wave may function as a positive regulator for inflammatory cytokines by enhancing NF-κB’s DNA-binding activity. These findings will help us understand the regulation and importance of intracellular Zn signaling in a variety of biological responses in which Zn-susceptible proteins are involved.

## Materials and Methods

### Cell Culture and Mice

All animal experiments were conducted in accordance with animal protocols approved by the Animal Research Committee at RIKEN (permit number 22-013(3)). Bone marrow-derived mast cells (BMMCs) were prepared as described previously [47]. Briefly, 8-week-old C57/BL6 mice were sacrificed, and their bone-marrow cells were cultured in RPMI 1640 supplemented with 10% FCS, 10 mU/mL penicillin, 0.1 mg/mL streptomycin, 40 µM 2-ME, and IL-3, in a 5% CO_2_ and 95% humidified atmosphere at 37°C. After 4–5 weeks of culture, the cell-surface expression of FcεRI and c-Kit was confirmed, and the cells were used for experiments.

### Reagents and Antibodies

Newport Green DCF diacetate, Fluo-4 AM, 4′,6-diamidino-2-phenylidole, dihydrochloride (DAPI), bis-(1,3-dibutylbarbituric acid) trimethine oxonol (DiBAC_4_(3)), and tetramethyl rhodamine methyl ester perchlorate (TMRM) were from Molecular Probes. Anti-Flag (M2) and anti-β-tubulin were from Sigma, anti-Cav1.3 polyclonal antibody was from Alomone Labs, anti-Cav1.3 monoclonal antibody L48A/9 was from NeuroMab, anti-GM130 was from BD Transduction Laboratories, anti-SERCA2 was from Abcam, anti-NF-κB p65 was from Santa Cruz Biotechnology, anti-LAT was from Upstate, and anti-phospho IKKα/β, anti-IKKα, anti-IκBα, and anti-phospho IκBα were from Cell Signaling. The siRNAs for control (siGENOME Non-Targeting siRNA Pool #1) and mouse *Cacna1d* (siGENOME SMARTpool, Mouse CACNA1D (12289)) were purchased from Thermo Fisher Scientific.

### Plasmid Construction and Transfection

The coding region of the human *CACNA1D* gene was isolated from a cDNA library of human brain (Clontech). To construct N-terminally FLAG-tagged *CACNA1D*, the CACNA1D fragment was amplified by PCR, followed by sequencing and cloning into the NotI and XbaI site of the expression vector p3XFLAG-Myc-CMV26 (Sigma Aldrich). BMMCs were transfected with expression vectors or siRNA using a two-step electroporator CUY21Pro-Vitro (Nepa Gene, Japan). For the electroporation, 1×10^6^ BMMCs were resuspended in 100 µl of OPTI-MEM, and 10 µg of plasmid DNA or 400 pmol of siRNA was added. Electroporation was carried out with 1 pore-forming pulse (275 V for 3 msec) and 10 driving pulses (20 V for 50 msec), and then the BMMCs were diluted in 1 ml of BMMC culture medium. After a 48-h incubation, the cells were used for experiments.

### RT-PCR and Real-time Quantitative RT-PCR Analyses

Total RNA was extracted and reverse-transcribed as previously described [32]. For standard RT-PCR, each sample was subjected to PCR with sense and antisense primers: *cacna1s*, 5′-TGTGGTATGTCGTCACTTCCTCC (sense) and 5′-CGTCAATGATGCTGCCGATG (antisense); *cacna1c*, 5′-CAAGCCCTCACAAAGGAATGC (sense) and 5′-AAAGTTGCCCCTGCTGTCACTC; *cacna1d*, 5′-ATCTCACACACCGCCAGGACTATG (sense) and 5′-CATCACCTTTGACCTCTCTCGTG (antisense); *cacna1f*, 5′-AAGATTTACCTATCCCAGGCACCTAC (sense) and 5′-CATCAAAGCGGGAAAGAATAGACTC (antisense). For real-time quantitative RT-PCR, the IL-6 and TNF-α gene expressions were measured relative to GAPDH using SYBR^®^ Green (Applied Biosystems). The primers used in these experiments were as follows: IL-6, 5′- GAGGATACCACTCCCAACAGACC (sense) and 5′- AAGTGCATCATCGTTGTTCATACA (antisense); TNF-α, 5′- CATCTTCTCAAAATTCGAGTGACAA (sense) and reverse primer, 5′- TGGGAGTAGACAAGGTACAACCC (antisense); GAPDH, 5′- TTCACCACCATGGAGAAGGCCG (sense) and 5′- GGCATGGACTGTGGTCATGA (antisense). The primer pairs used to detect *Cacna1* family members were the Perfect Real Time Primer (Takara Bio Inc, Japan) for the *Mus musculus* calcium channel, voltage-dependent, alpha 1S subunit (Cacna1s), transcript variant 2 mRNA (Position 5019), *M. musculus* alpha 1C subunit (Cacna1c) mRNA (Position 5787), *M. musculus* alpha 1D subunit (Cacna1d), transcript variant 2 mRNA (Position 6650), and *M. musculus* alpha 1F subunit (Cacna1f) mRNA (Position 5601).

### Cell Lysates and Immunoblotting

BMMCs were harvested, lysed with lysis buffer (20 mM Tris-HCl pH 7.4, 150 mM NaCl, 1% NP-40, proteinase inhibitors, 5 µg/ml pepstatin, 10 µg/ml leupeptin) for 30 min at 4°C, and spun at 12,000×g, 4°C, for 30 min. The eluted and reduced samples were resolved by SDS-PAGE using a 4–20% gradient polyacrylamide gel (Wako), and transferred to a PVDF membrane (Immobilon-P, Millipore). For immunoblotting, the membranes were incubated with the primary antibodies anti-Cav1.3 (1∶500), anti-LAT (1∶1000), anti-SERCA2 (1∶1000), anti-phospho IκBα (1∶2000), anti-IκBα (1∶1000), anti-β-tubulin (1∶5000), anti-phospho IKKα/β (1∶1000), or anti-IKKα (1∶500) antibodies. The membranes were then incubated with HRP-conjugated anti-mouse, rabbit, or goat IgG (Zymed) for 1 h at room temperature. After extensive washing of the membranes, immunoreactive proteins were visualized using the Western Lightning-ECL system, according to the manufacturer’s recommendation. The PVDF membranes were exposed to Fuji RX film (Fuji). Densitometric analysis was performed using an LAS-1000 fluorescence image analyzer (Fujifilm).

### Microscopy

The intracellular Zn or Ca^2+^ level was measured as described previously [9]. Briefly, sensitized BMMCs were allowed to adhere to a poly-L-lysine-coated glass-bottom dish or glass-bottom dish. After being incubated with 10 µM Newport Green or 5 µM Fluo-4 for 30 min at 37°C, the cells were stimulated with 100 ng/ml dinitrophenylated human serum albumin (DNP-HSA; Sigma) or 10 µM (s)-(-)-BayK8644 at 37°C. The images of fluorescent signals were captured every 10 or 30 sec with an inverted microscope (Axiovert 200 MOT, Carl Zeiss), CCD camera (Cool Snap HQ, Roper Scientific), and the system control application SlideBook (Intelligent Imaging Innovation).

For the immunostaining of BMMCs, the cells on a poly-L-lysine coated dish were fixed with 4% paraformaldehyde for 10 min at 37°C, then permeabilized in Perm Buffer (BD) containing 1% BSA for 15 min at room temperature. Primary and secondary staining were performed on the poly-L-lysine coated dish: anti-Cav1.3 at a dilution of 1∶50, anti-SERCA2 at 1∶100, anti-p65 at 1∶50, Alexa488-conjugated anti-rabbit IgG, (Molecular Probes), Phalloidin-Rhodamine (Molecular Probes) at 1∶100, and DAPI at 1∶5000. Confocal microscopy was carried out using the TCS SL system (Leica). Images were transferred to Adobe Photoshop CS3.

### Sucrose Gradient Fractionation Assay

Mature BMMCs (5×10^7^) were harvested and washed with PBS and homogenized with a Dounce homogenizer in 0.5 ml of ice-chilled HES buffer (250 mM sucrose, 1 mM EDTA, 20 mM HEPES, pH 7.5). The homogenate was spun at 500×g for 5 min to sediment the nuclei, and the postnuclear supernatant was used for further fractionation. The postnuclear supernatant was mixed with 0.5 ml of 0.8 M sucrose in 50 mM Tris-HCl and loaded onto the top of a discontinuous sucrose gradient (0.6, 1, 1.35, 1.65, 2 M) prepared in the same buffer. The gradient was spun in a SW 55 Ti rotor for 16 h at 100,000×g (32,100 rpm) in a Beckman ultracentrifuge, and fractions of 300 µl each were collected from the top of the tube. Proteins from each fraction were separated by SDS-PAGE.

### Measurement of Cytokines

Cells were sensitized with 1 mg/mL IgE for 6 h at 37°C. After sensitization, the cells were washed twice with Tyrode’s buffer (10 mM HEPES pH 7.4, 130 mM NaCl, 5 mM KCl, 1.4 mM CaCl_2_, 1 mM MgCl_2_, 5.6 mM glucose), then suspended in the same buffer containing 0.1% BSA and stimulated with polyvalent dinitrophenyl-human serum albumin (DNP-HSA, Sigma) for 30 min. TNF-α and IL-6 in the cell culture supernatants were measured with an ELISA kit (Biosource), following the manufacturer’s recommendation.

### BMMC Degranulation Assay

Cells were activated as described above, then spun down at 6000×g for 1 min, and 50 µl of the culture supernatant or the cell pellet solubilized with 1% Triton X-100 in Tyrode’s buffer was combined with 100 µL of 1.3 mg/mL p-nitrophenyl-N-acetyl-D-glucosamide and developed for 60 min at 37°C. The enzyme reaction was stopped by adding 150 µL of 0.2 M glycine-NaOH (pH 10.2), and the absorbance at 405 nm was measured with a microplate reader (Bio-Rad).

### FITC-induced Contact Hypersensitivity

The FITC-induced CHS procedure was performed as described previously [46]. Briefly, mice were sensitized by applying 200 µl of 2% FITC isomer-I (FITC; Sigma-Aldrich) in a vehicle consisting of acetone-dibutylphthalate (1∶1) to the skin of the back. Five days after the sensitization with FITC, the mice were pretreated with 20 µl of acetone-EtOH (1∶1) or 50 mg/ml Verapamil in acetone-EtOH (1∶1) and then challenged with 20 µl of vehicle alone on the right ear (10 µl on each side of the ear) and 1% FITC on the left ear (10 µl on each side). The ear thickness was measured before and at various times after FITC challenge, with an engineer’s microcaliper (Ozaki).

### Passive Cutaneous Anaphylaxis

A total of 2 µg IgE in 20 µl was injected subcutaneously into the ears over a period of 12 h. After the sensitization, the mice were challenged with an intravenous injection of 50 µl polyvalent dinitrophenyl-bovine serum albumin (DNP-BSA: Cosmobio, Japan) in 250 µl of saline-5 mg/mL Evans blue dye (Sigma, Japan). The extravasation of Evans blue into the ear was monitored for 30 min. The mice were then sacrificed, both ears were dissected, and the Evans blue dye was extracted in 700 µl of formamide at 63°C overnight. The absorbance of the Evans blue-containing formamide was measured at 620 nm.

### NF-κB DNA-binding Assay

The DNA-binding activity of NF-κB p65 was examined with the TransAM™ NFκB p65 kit (Active Motifs), according to the manufacturer’s protocol. In brief, sensitized BMMCs (2×10^6^) were rinsed twice with Tyrode’s buffer and incubated with or without 100 µM Verapamil for 30 min at 37°C, then stimulated with 10 ng/ml DNP-HSA for 30 min at 37°C with or without 1 µM pyrithione and ZnSO_4_. The cytosolic and nuclear proteins were separated using the Nuclear Extraction Kit (TransAM) according to the manufacturer’s protocol. Nuclear proteins were prepared in a 50 µl volume, and 20 µl of each sample was used to estimate the amount of DNA-bound NF-κB p65, and 15 µl of each sample was subjected to SDS-PAGE to determine the total amount of NF-κB protein in the nuclear fraction. The DNA-binding activity of the NF-κB p65 in the nuclear fraction was estimated by dividing the amount of NF-κB p65 bound to the target sequence by the amount of NF-κB p65 protein in the nuclear fraction.

### Statistical Analysis

All statistical analyses were performed using Statcel software. Data were analyzed by two-tailed Student’s t-test or Student’s t-test with Bonferroni’s correction for multiple comparison. Data were considered statistically significant when the *P* value was less than 0.05. N.S., not significant, *P<0.05, **P<0.01, ***P<0.001.

## Supporting Information

Figure S1 **Effect of the LTCC antagonist Verapamil on cell survival and the surface expression of receptors.** (A) BMMC survival after a 3-h treatment with the indicated concentrations of Verapamil was determined by flow cytometry. Cell viability was detected by staining BMMCs with 7-AAD. Numbers show the percentage of total cells that were 7-AAD-positive dead cells. Zn toxicity-induced cell death was observed as a control by treating the cells with 1 µM pyrithione and 10 µM ZnSO_4_ for 3 h. (B) The surface expression levels of c-kit and FcεRI were examined in BMMCs that were untreated or treated with 50 or 100 µM Verapamil for 3 h.
(TIF)

Figure S2 **LTCC antagonists inhibit the FcεRI-mediated Zn wave.** (A) The intracellular labile Zn level after FcεRI-mediated stimulation was examined using the fluorescent Zn indicator Newport Green in mast cells with or without pre-treatment with 1 µM Verapamil. The data represent the relative fluorescent intensity of Newport Green, means + SEM. The difference in Newport Green intensity at 15 min between the control and Verapamil-treated BMMCs was statistically significant. *P<0.05, Student’s *t*-test. (B) The intracellular labile Zn level after FcεRI-mediated stimulation was examined in mast cells with or without pre-treatment with 100 µM (+)-cis-Diltiazem hydrochloride (Sigma Aldrich). The data represent the relative fluorescent intensity of Newport Green, means + SEM. The difference in Newport Green intensity at 15 min between the control and Diltiazem-treated BMMCs was statistically significant. *P<0.05. (C) The FcεRI-mediated Ca^2+^ elevation in control and Diltiazem-treated BMMCs was examined using the fluorescent Ca^2+^ indicator Fluo-4. Data represent the relative fluorescent intensity of Fluo-4, means + SEM. The difference in Fluo-4 intensity between the control and Diltiazem-treated BMMCs was not statistically significant, Student’s *t*-test. All data are representative of at least three experiments.
(TIF)

Figure S3 **An LTCC agonist can induce the Zn wave without antigen stimulation.** (A) Time-lapse recording of the Newport Green signal in BMMCs treated with 10 µM (s)-(-)-BayK8644, an LTCC agonist. Images were converted into pseudocolors, in which low intensity is blue and high intensity is red. (B) The intracellular Ca^2+^ elevation upon agonist treatment was examined using the fluorescent Ca^2+^ indicator Fluo-4. After 10 min of (s)-(-)-BayK8644 treatment, 1 µM ionomycin, a Ca^2+^ ionophore, was added to the buffer and the intracellular Ca^2+^ was further examined for 150 seconds. (C) The intracellular Zn level in BMMCs treated with the indicated concentrations of (s)-(-)-BayK8644 for 15 min in control or Ca^2+^-free Tyrode’s buffer was determined by flow cytometry. (D) The intracellular Zn level in BMMCs with or without (s)-(-)-BayK8644 treatment along with antigen stimulation was determined by flow cytometry. BMMCs were stimulated with 100 ng/ml DNP-HSA and 5 µM (s)-(-)-BayK8644 for 15 min. NPG, Newport Green; Bay, (s)-(-)-BayK8644.
(TIF)

Figure S4 **Introduction of siRNA for α_1D_ into BMMCs.** (A) The mRNA levels of *Cacna1s* were determined by semi-quantitative RT-PCR in control (siControl) or α_1D_ siRNA-treated (si α_1D_) BMMCs. Under our experimental conditions, *Cacna1s* and *Cacna1c* were below the detection level. The mRNA level in si α_1D_–treated BMMCs was 24.5±5.2% (for *Cacna1d*) and 98.6±12.8% (for *Cacna1f*) of that in control cells. Data are representative of three independent experiments. (B) The protein level of α_1D_ was examined in control and α_1D_ siRNA-treated BMMCs. Arrowhead indicates the putative α_1D_ signal. The protein level in si α_1D_–treated BMMCs was 32.6±10.5% of that in control cells.
(TIF)

Figure S5 **Effect of an exportin inhibitor on the inhibition of nuclear translocation by an LTCC antagonist.** (A) The frequency of NF-κB nuclear-translocated cells was determined by confocal microscopy. BMMCs were pretreated with 20 ng/ml leptomycin B for 2 h, and 100 µM Verapamil, an LTCC antagonist, for 30 min, then stimulated with 100 ng/ml DNP-HSA for 15 min. (B) ZnSO_4_ at the indicated concentration was added to the cytoplasmic compartment of BMMCs for 30 min, and then the DNA-binding activity of NF-κB was determined.
(TIF)

Figure S6 **Effect of Diltiazem on allergic responses in vivo.** Analysis of the effect of Diltiazem on contact hypersensitivity. Vehicle-, Verapamil-, or Diltiazem-treated mice were sensitized with FITC, and ear swelling was measured at the indicated times after hapten challenge. Data represent means + S.E.M. (n = 6 for vehicle, n = 4 for Verapamil, and n = 5 for Diltiazem from two independently performed experiments). *P<0.05, Bonferroni’s multiple comparison test.
(TIF)

Figure S7 **Effect of Verapamil on FcεRI-mediated Ca^2+^ signaling.** NFAT2 nuclear translocation was visualized by confocal microscopy. BMMCs were untreated or treated with Verapamil and stimulated with antigen for 30 min. The frequency of NFAT2 nuclear-translocated cells was determined. Data represent the means + S.D. N.S., not significant, Bonferroni’s multiple comparison test.
(TIF)

Figure S8 **Effect of membrane potential changes on Zn-wave generation.** (A) The plasma membrane potential was determined using a membrane potential-sensitive fluorescent dye DiBAC_4_(3) and flow cytometry. Data represent the mean fluorescent intensity of DiBAC_4_(3). (B) The effect of a high concentration of KCl on the FcεRI-mediated Zn wave was determined by examining the intracellular labile Zn level with or without 50 mM KCl treatment. Data represent the relative fluorescent intensity of Newport Green. (C) The intracellular membrane potential was determined with the cationic dye TMRM. BMMCs were treated with 20 µM Bongkrekic acid for 30 min before stimulation. The TMRM intensity of antigen-stimulated cells was determined by flow cytometry. Data represent the relative fluorescent intensity of TMRM. (D) The intracellular Zn level of BMMCs treated with or without 20 µM Bongkrekic acid upon antigen stimulation was determined by flow cytometry. Data represent the relative fluorescent intensity of Newport Green. N.S., not significant, *P<0.05, **P<0.01. Ag, Antigen; NPG, Newport Green.
(TIF)

Figure S9 **Effect of PKA-mediated signaling on the FcεRI-induced Zn wave.** The FcεRI-mediated Zn wave was examined in BMMCs treated with a PKA inhibitor at the indicated concentration. The intracellular labile Zn level was determined by staining the BMMCs with Newport Green and analyzed by flow cytometry. Data represent the mean fluorescent intensity of Newport Green ± S.D. ***P<0.001 Student’s t-test, two-tailed. NPG, Newport Green.
(TIF)

Figure S10 **Effect of LTCC agonist-induced Zn elevation on cytokine production.** The production of IL-6 and TNF-α upon antigen stimulation for 3 hours was measured by ELISA in BMMCs with or without pre-treatment with 20 µM (s)-(-)-BayK8644, an LTCC agonist in (A) and (C). FcεRI-mediated induction of *Il6* and *Tnfa* transcription upon antigen stimulation in BMMCs with or without treatment with 20 µM (s)-(-)-BayK8644 was determined. The mRNA levels of *Il6* and *Tnfa* were determined by semi-quantitative RT-PCR analysis in (s)-(-)-BayK8644-treated cells (B) and (D). N.S., not significant, Student’s *t*-test, two-tailed. Bay, (s)-(-)-BayK8644.
(TIF)

## References

[1] Andreini C, Banci L, Bertini I, Rosato A (2006). Counting the zinc-proteins encoded in the human genome.. J Proteome Res.

[2] Hambidge M (2000). Human zinc deficiency.. J Nutr.

[3] Prasad AS (2001). Recognition of zinc-deficiency syndrome.. Nutrition.

[4] Maret W (2011). Metals on the move: zinc ions in cellular regulation and in the coordination dynamics of zinc proteins.. Biometals.

[5] Hirano T, Murakami M, Fukada T, Nishida K, Yamasaki S (2008). Roles of zinc and zinc signaling in immunity: zinc as an intracellular signaling molecule.. Adv Immunol.

[6] Frederickson CJ, Bush AI (2001). Synaptically released zinc: physiological functions and pathological effects.. Biometals.

[7] Frederickson CJ, Koh JY, Bush AI (2005). The neurobiology of zinc in health and disease.. Nat Rev Neurosci.

[8] Sensi SL, Paoletti P, Bush AI, Sekler I (2009). Zinc in the physiology and pathology of the CNS.. Nat Rev Neurosci.

[9] Yamasaki S, Sakata-Sogawa K, Hasegawa A, Suzuki T, Kabu K (2007). Zinc is a novel intracellular second messenger.. J Cell Biol.

[10] Murakami M, Hirano T (2008). Intracellular zinc homeostasis and zinc signaling.. Cancer Sci.

[11] Fukada T, Yamasaki S, Nishida K, Murakami M, Hirano T (2011). Zinc homeostasis and signaling in health and diseases: Zinc signaling.. J Biol Inorg Chem.

[12] Fukada T, Civic N, Furuichi T, Shimoda S, Mishima K (2008). The zinc transporter SLC39A13/ZIP13 is required for connective tissue development; its involvement in BMP/TGF-beta signaling pathways.. PLoS One.

[13] Kitamura H, Morikawa H, Kamon H, Iguchi M, Hojyo S (2006). Toll-like receptor-mediated regulation of zinc homeostasis influences dendritic cell function.. Nat Immunol.

[14] Yamashita S, Miyagi C, Fukada T, Kagara N, Che YS (2004). Zinc transporter LIVI controls epithelial-mesenchymal transition in zebrafish gastrula organizer.. Nature.

[15] Hojyo S, Fukada T, Shimoda S, Ohashi W, Bin BH (2011). The zinc transporter SLC39A14/ZIP14 controls G-protein coupled receptor-mediated signaling required for systemic growth.. PLoS One.

[16] Nishida K, Hasegawa A, Nakae S, Oboki K, Saito H (2009). Zinc transporter Znt5/Slc30a5 is required for the mast cell-mediated delayed-type allergic reaction but not the immediate-type reaction.. J Exp Med.

[17] Haase H, Hebel S, Engelhardt G, Rink L (2006). Flow cytometric measurement of labile zinc in peripheral blood mononuclear cells.. Anal Biochem.

[18] Haase H, Ober-Blobaum JL, Engelhardt G, Hebel S, Heit A (2008). Zinc signals are essential for lipopolysaccharide-induced signal transduction in monocytes.. J Immunol.

[19] Yu M, Lee WW, Tomar D, Pryshchep S, Czesnikiewicz-Guzik M (2011). Regulation of T cell receptor signaling by activation-induced zinc influx.. J Exp Med.

[20] Atar D, Backx PH, Appel MM, Gao WD, Marban E (1995). Excitation-transcription coupling mediated by zinc influx through voltage-dependent calcium channels.. J Biol Chem.

[21] Gyulkhandanyan AV, Lee SC, Bikopoulos G, Dai F, Wheeler MB (2006). The Zn2+-transporting pathways in pancreatic beta-cells: a role for the L-type voltage-gated Ca2+ channel.. J Biol Chem.

[22] Sensi SL, Canzoniero LM, Yu SP, Ying HS, Koh JY (1997). Measurement of intracellular free zinc in living cortical neurons: routes of entry.. J Neurosci.

[23] Catterall WA (2000). Structure and regulation of voltage-gated Ca2+ channels.. Annu Rev Cell Dev Biol.

[24] Bichet D, Cornet V, Geib S, Carlier E, Volsen S (2000). The I-II loop of the Ca2+ channel alpha1 subunit contains an endoplasmic reticulum retention signal antagonized by the beta subunit.. Neuron.

[25] Cornet V, Bichet D, Sandoz G, Marty I, Brocard J (2002). Multiple determinants in voltage-dependent P/Q calcium channels control their retention in the endoplasmic reticulum.. Eur J Neurosci.

[26] Baeuerle PA, Henkel T (1994). Function and activation of NF-kappa B in the immune system.. Annu Rev Immunol.

[27] Karin M, Lin A (2002). NF-kappa B at the crossroads of life and death.. Nat Immunol.

[28] Karin M, Ben-Neriah Y (2000). Phosphorylation meets ubiquitination: The control of NF-kappa B activity.. Annu Rev Immunol 18: 621–+.

[29] Metcalfe DD, Baram D, Mekori YA (1997). Mast cells.. Physiol Rev.

[30] Galli SJ, Kalesnikoff J, Grimbaldeston MA, Piliponsky AM, Williams CMM (2005). Mast cells as “tunable” effector and immunoregulatory cells: Recent advances.. Annu Rev Immunol.

[31] Pahl HL (1999). Activators and target genes of Rel/NF-kappa B transcription factors.. Oncogene.

[32] Kabu K, Yamasaki S, Kamimura D, Ito Y, Hasegawa A (2006). Zinc is required for FcepsilonRI-mediated mast cell activation.. J Immunol.

[33] Okamoto T, Ogiwara H, Hayashi T, Mitsui A, Kawabe T (1992). Human thioredoxin/adult T cell leukemia-derived factor activates the enhancer binding protein of human immunodeficiency virus type 1 by thiol redox control mechanism.. Int Immunol.

[34] Toledano MB, Ghosh D, Trinh F, Leonard WJ (1993). N-terminal DNA-binding domains contribute to differential DNA-binding specificities of NF-kappa B p50 and p65.. Mol Cell Biol.

[35] Yang JP, Merin JP, Nakano T, Kato T, Kitade Y (1995). Inhibition of the DNA-binding activity of NF-kappa B by gold compounds in vitro.. FEBS Lett.

[36] Kotturi MF, Hunt SV, Jefferies WA (2006). Roles of CRAC and Cav-like channels in T cells: more than one gatekeeper?. Trends Pharmacol Sci.

[37] Marquardt DL, Walker LL (2000). Dependence of mast cell IgE-mediated cytokine production on nuclear factor-kappaB activity.. J Allergy Clin Immunol.

[38] Levy S, Beharier O, Etzion Y, Mor M, Buzaglo L (2009). Molecular basis for zinc transporter 1 action as an endogenous inhibitor of L-type calcium channels.. J Biol Chem.

[39] Vig M, Kinet JP (2009). Calcium signaling in immune cells.. Nat Immunol.

[40] Vennekens R, Olausson J, Meissner M, Bloch W, Mathar I (2007). Increased IgE-dependent mast cell activation and anaphylactic responses in mice lacking the calcium-activated nonselective cation channel TRPM4.. Nat Immunol.

[41] Shumilina E, Lam RS, Wolbing F, Matzner N, Zemtsova IM (2008). Blunted IgE-mediated activation of mast cells in mice lacking the Ca2+-activated K+ channel KCa3.1.. J Immunol.

[42] Ramadan O, Qu Y, Wadgaonkar R, Baroudi G, Karnabi E (2009). Phosphorylation of the consensus sites of protein kinase A on alpha1D L-type calcium channel.. J Biol Chem.

[43] Klemm S, Gutermuth J, Hultner L, Sparwasser T, Behrendt H (2006). The Bcl10-Malt1 complex segregates Fc epsilon RI-mediated nuclear factor kappa B activation and cytokine production from mast cell degranulation.. J Exp Med.

[44] Biedermann T, Kneilling M, Mailhammer R, Maier K, Sander CA (2000). Mast cells control neutrophil recruitment during T cell-mediated delayed-type hypersensitivity reactions through tumor necrosis factor and macrophage inflammatory protein 2.. J Exp Med.

[45] Kakurai M, Monteforte R, Suto H, Tsai M, Nakae S (2006). Mast cell-derived tumor necrosis factor can promote nerve fiber elongation in the skin during contact hypersensitivity in mice.. Am J Pathol.

[46] Suto H, Nakae S, Kakurai M, Sedgwick JD, Tsai M (2006). Mast cell-associated TNF promotes dendritic cell migration.. J Immunol.

[47] Nishida K, Wang L, Morii E, Park SJ, Narimatsu M (2002). Requirement of Gab2 for mast cell development and KitL/c-Kit signaling.. Blood.

