# Supplementary figures and images for: A Novel Role of the L-Type Calcium Channel α1D Subunit as a Gatekeeper for Intracellular Zinc Signaling: Zinc Wave
Source: PLoS One. 2012 Jun 22;7(6):e39654. doi: 10.1371/journal.pone.0039654 (PMC3382136; doi:10.1371/journal.pone.0039654)

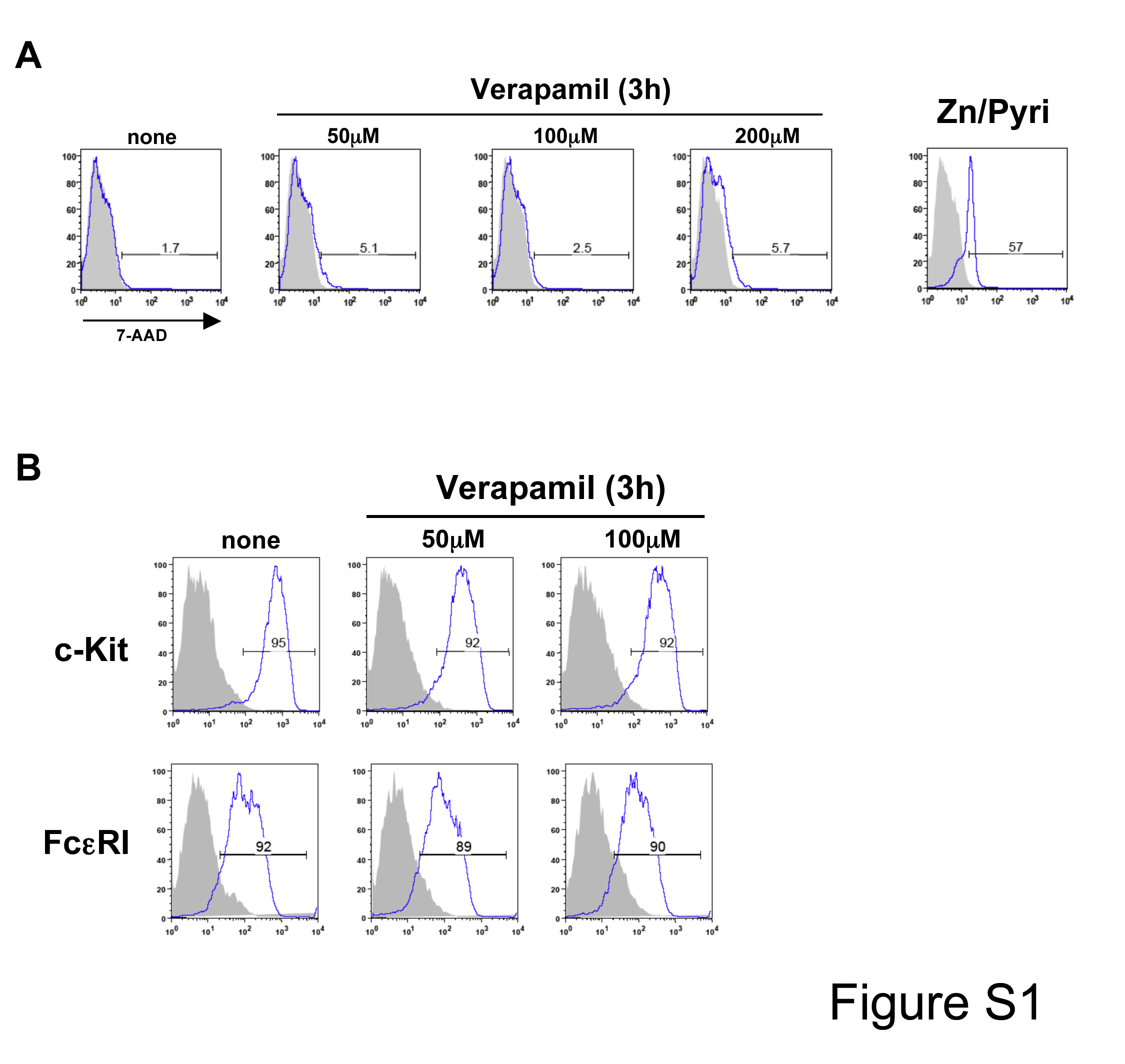

Supplement: Figure S1 — Effect of the LTCC antagonist Verapamil on cell survival and the surface expression of receptors. (A) BMMC survival after a 3-h treatment with the indicated concentrations of Verapamil was determined by flow cytometry. Cell viability was detected by staining BMMCs with 7-AAD. Numbers show the percentage of total cells that were 7-AAD-positive dead cells. Zn toxicity-induced cell death was observed as a control by treating the cells with 1 µM pyrithione and 10 µM ZnSO4 for 3 h. (B) The surface expression levels of c-kit and FcεRI were examined in BMMCs that were untreated or treated with 50 or 100 µM Verapamil for 3 h. (TIF) [file pone.0039654.s001.tif]

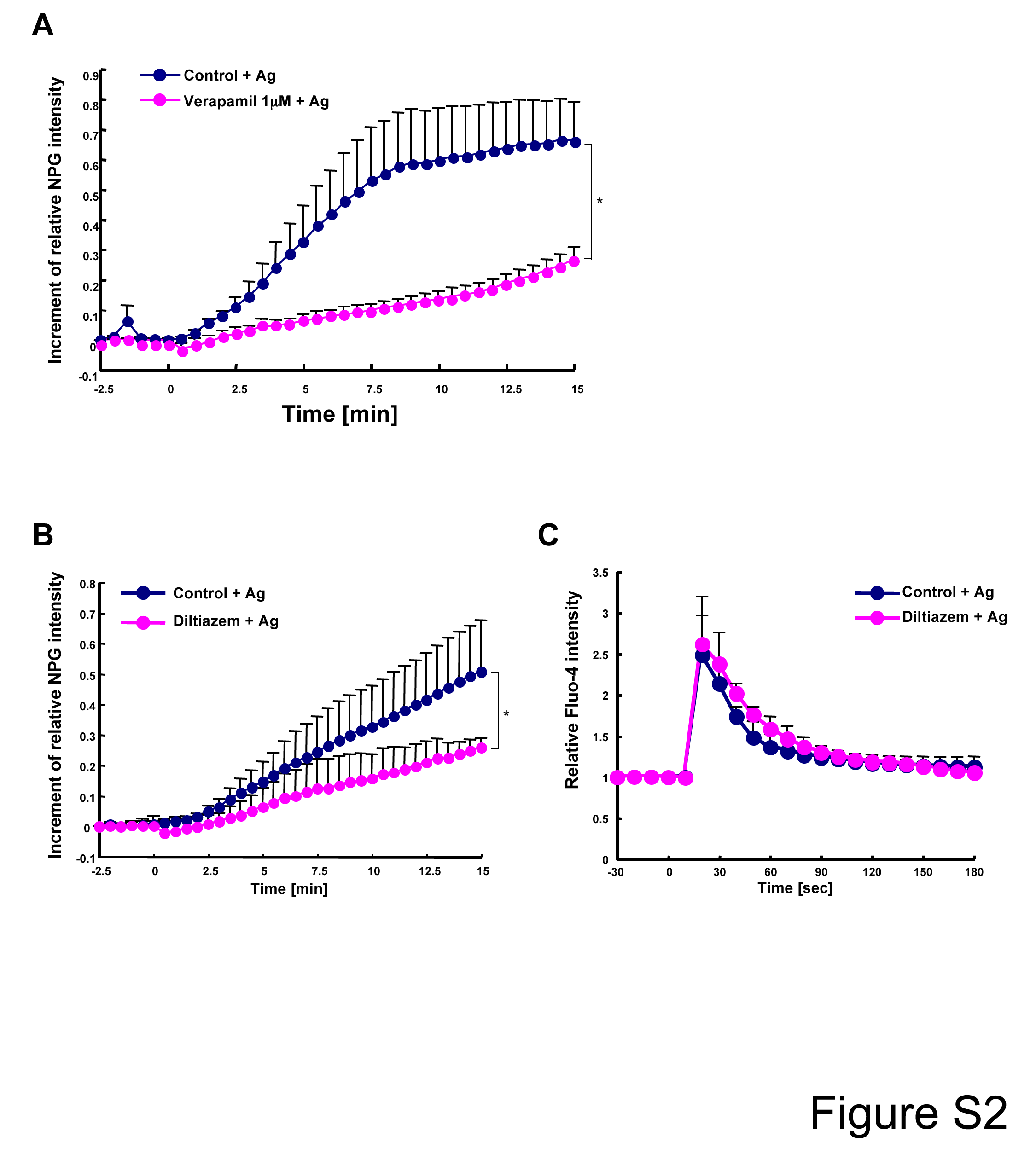

Supplement: Figure S2 — LTCC antagonists inhibit the FcεRI-mediated Zn wave. (A) The intracellular labile Zn level after FcεRI-mediated stimulation was examined using the fluorescent Zn indicator Newport Green in mast cells with or without pre-treatment with 1 µM Verapamil. The data represent the relative fluorescent intensity of Newport Green, means + SEM. The difference in Newport Green intensity at 15 min between the control and Verapamil-treated BMMCs was statistically significant. *P<0.05, Student’s t-test. (B) The intracellular labile Zn level after FcεRI-mediated stimulation was examined in mast cells with or without pre-treatment with 100 µM (+)-cis-Diltiazem hydrochloride (Sigma Aldrich). The data represent the relative fluorescent intensity of Newport Green, means + SEM. The difference in Newport Green intensity at 15 min between the control and Diltiazem-treated BMMCs was statistically significant. *P<0.05. (C) The FcεRI-mediated Ca2+ elevation in control and Diltiazem-treated BMMCs was examined using the fluorescent Ca2+ indicator Fluo-4. Data represent the relative fluorescent intensity of Fluo-4, means + SEM. The difference in Fluo-4 intensity between the control and Diltiazem-treated BMMCs was not statistically significant, Student’s t-test. All data are representative of at least three experiments. (TIF) [file pone.0039654.s002.tif]

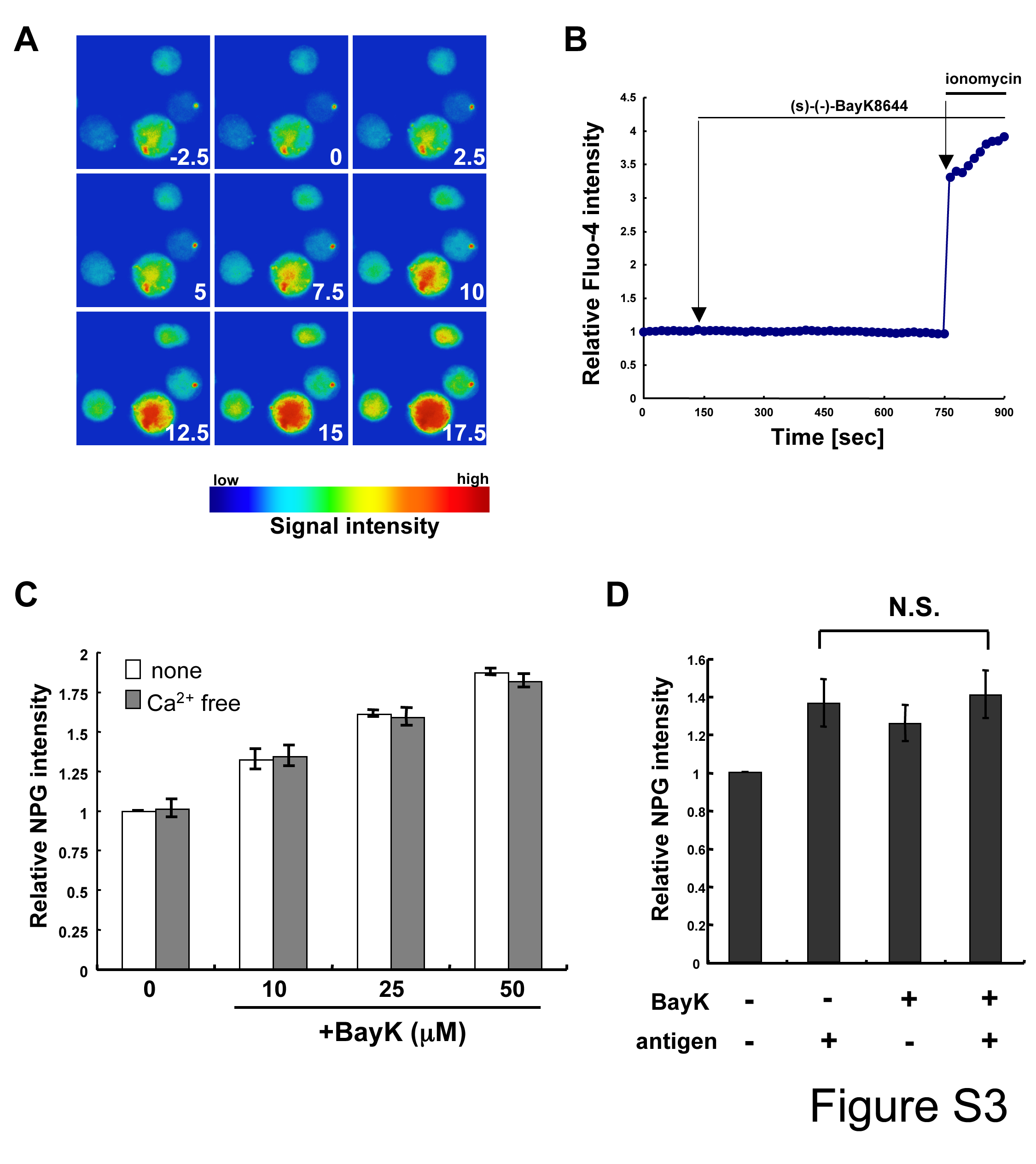

Supplement: Figure S3 — An LTCC agonist can induce the Zn wave without antigen stimulation. (A) Time-lapse recording of the Newport Green signal in BMMCs treated with 10 µM (s)-(-)-BayK8644, an LTCC agonist. Images were converted into pseudocolors, in which low intensity is blue and high intensity is red. (B) The intracellular Ca2+ elevation upon agonist treatment was examined using the fluorescent Ca2+ indicator Fluo-4. After 10 min of (s)-(-)-BayK8644 treatment, 1 µM ionomycin, a Ca2+ ionophore, was added to the buffer and the intracellular Ca2+ was further examined for 150 seconds. (C) The intracellular Zn level in BMMCs treated with the indicated concentrations of (s)-(-)-BayK8644 for 15 min in control or Ca2+-free Tyrode’s buffer was determined by flow cytometry. (D) The intracellular Zn level in BMMCs with or without (s)-(-)-BayK8644 treatment along with antigen stimulation was determined by flow cytometry. BMMCs were stimulated with 100 ng/ml DNP-HSA and 5 µM (s)-(-)-BayK8644 for 15 min. NPG, Newport Green; Bay, (s)-(-)-BayK8644. (TIF) [file pone.0039654.s003.tif]

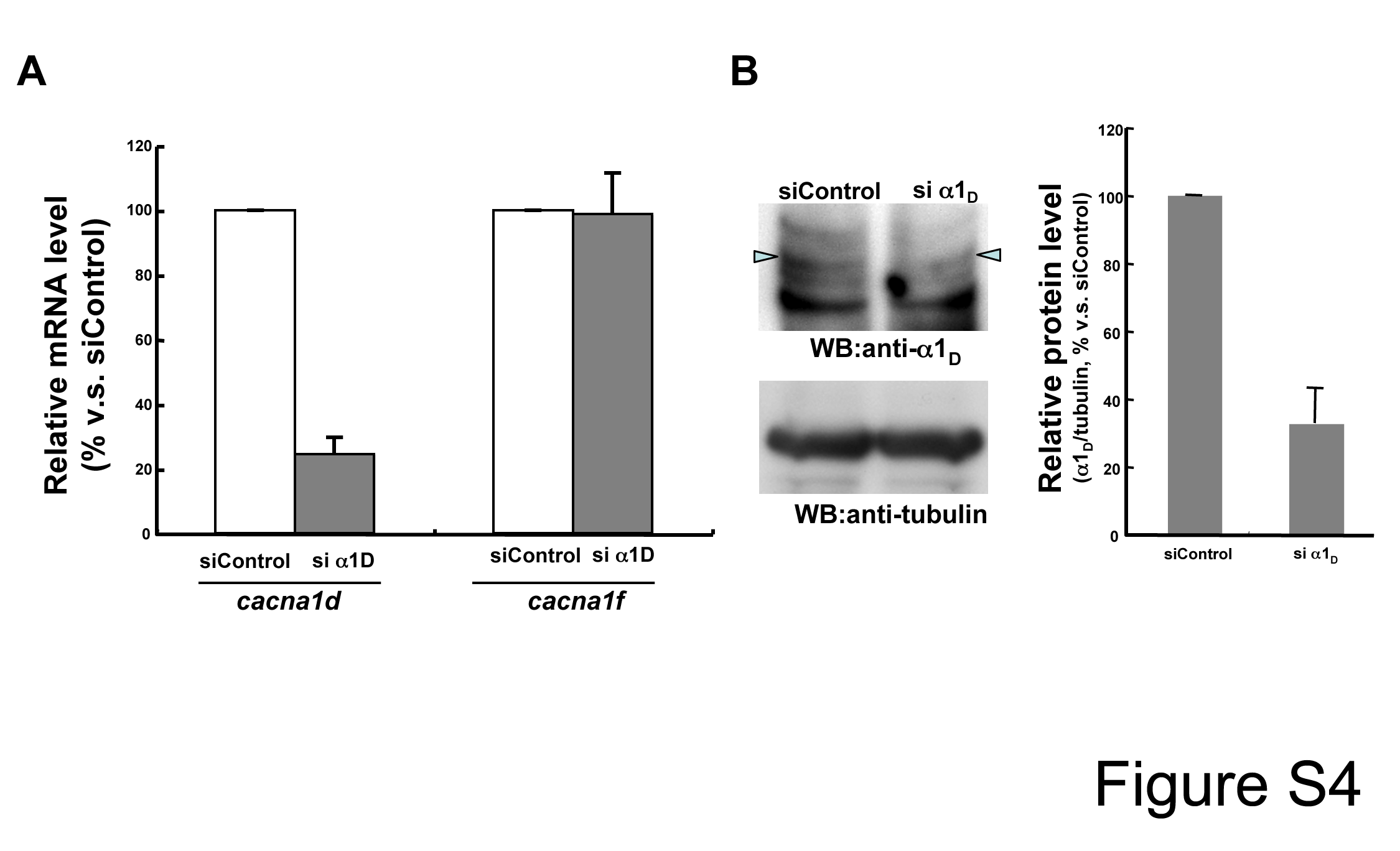

Supplement: Figure S4 — Introduction of siRNA for α1D into BMMCs. (A) The mRNA levels of Cacna1s were determined by semi-quantitative RT-PCR in control (siControl) or α1D siRNA-treated (si α1D) BMMCs. Under our experimental conditions, Cacna1s and Cacna1c were below the detection level. The mRNA level in si α1D–treated BMMCs was 24.5±5.2% (for Cacna1d) and 98.6±12.8% (for Cacna1f) of that in control cells. Data are representative of three independent experiments. (B) The protein level of α1D was examined in control and α1D siRNA-treated BMMCs. Arrowhead indicates the putative α1D signal. The protein level in si α1D–treated BMMCs was 32.6±10.5% of that in control cells. (TIF) [file pone.0039654.s004.tif]

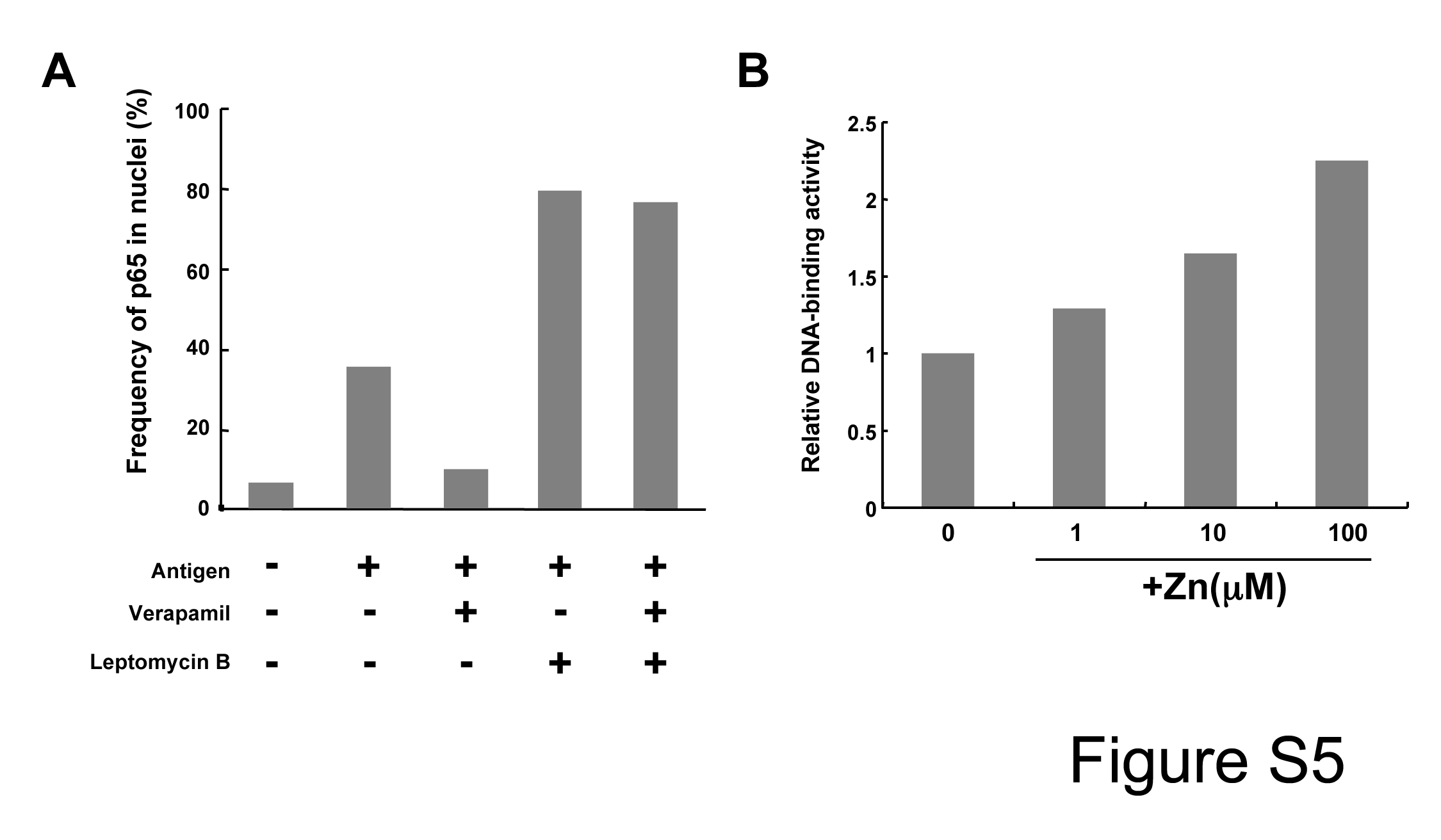

Supplement: Figure S5 — Effect of an exportin inhibitor on the inhibition of nuclear translocation by an LTCC antagonist. (A) The frequency of NF-κB nuclear-translocated cells was determined by confocal microscopy. BMMCs were pretreated with 20 ng/ml leptomycin B for 2 h, and 100 µM Verapamil, an LTCC antagonist, for 30 min, then stimulated with 100 ng/ml DNP-HSA for 15 min. (B) ZnSO4 at the indicated concentration was added to the cytoplasmic compartment of BMMCs for 30 min, and then the DNA-binding activity of NF-κB was determined. (TIF) [file pone.0039654.s005.tif]

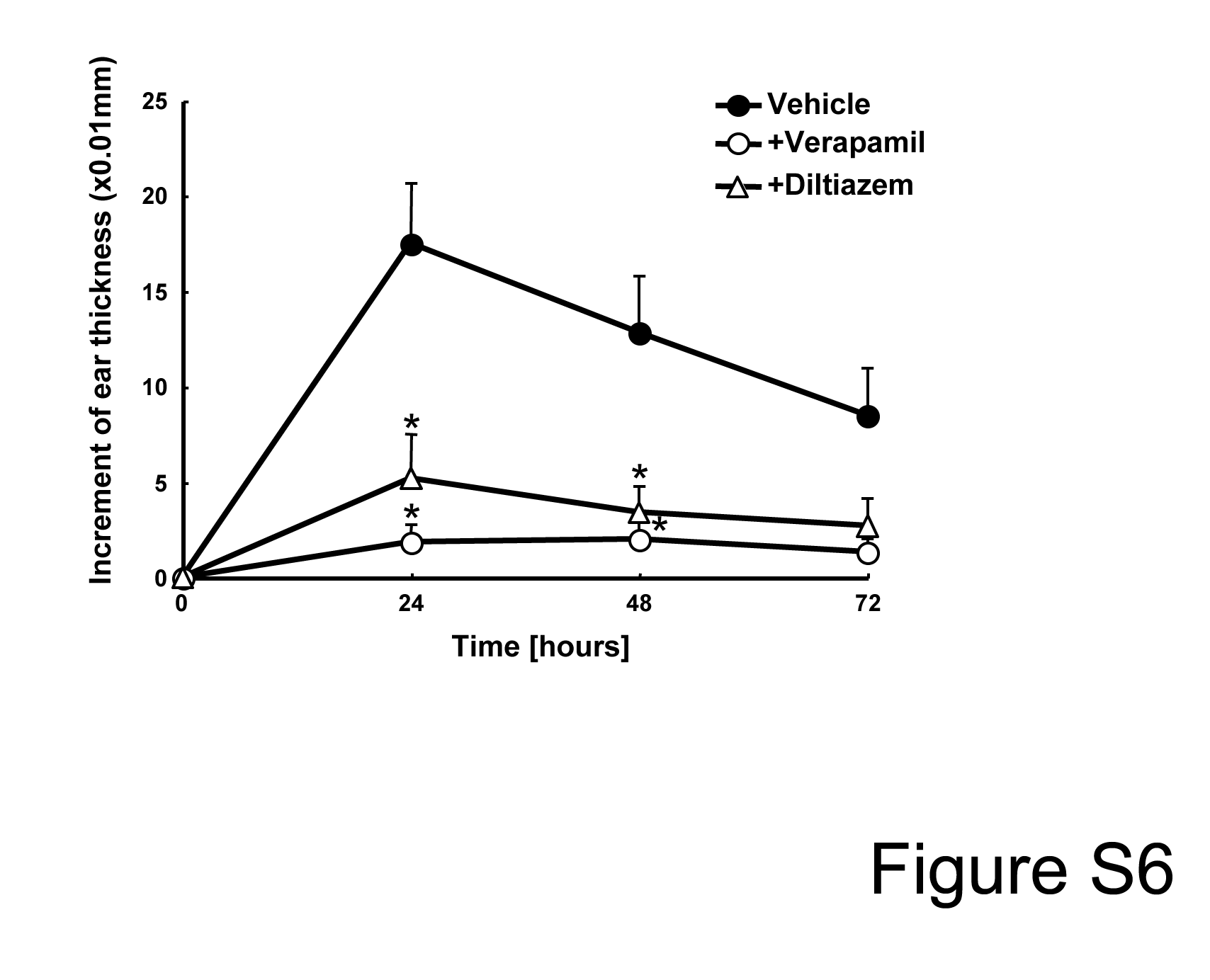

Supplement: Figure S6 — Effect of Diltiazem on allergic responses in vivo. Analysis of the effect of Diltiazem on contact hypersensitivity. Vehicle-, Verapamil-, or Diltiazem-treated mice were sensitized with FITC, and ear swelling was measured at the indicated times after hapten challenge. Data represent means + S.E.M. (n = 6 for vehicle, n = 4 for Verapamil, and n = 5 for Diltiazem from two independently performed experiments). *P<0.05, Bonferroni’s multiple comparison test. (TIF) [file pone.0039654.s006.tif]

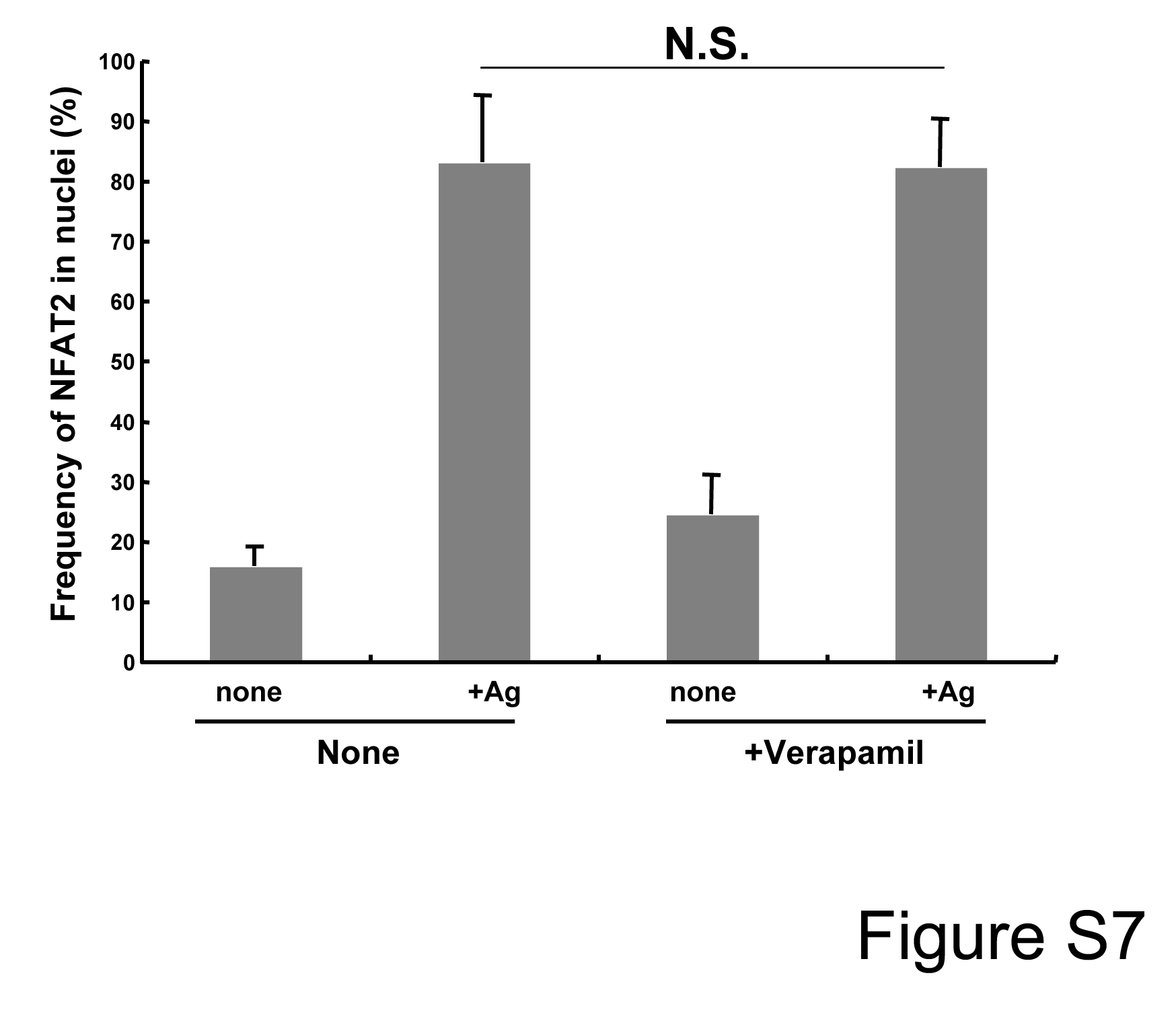

Supplement: Figure S7 — Effect of Verapamil on FcεRI-mediated Ca2+ signaling. NFAT2 nuclear translocation was visualized by confocal microscopy. BMMCs were untreated or treated with Verapamil and stimulated with antigen for 30 min. The frequency of NFAT2 nuclear-translocated cells was determined. Data represent the means + S.D. N.S., not significant, Bonferroni’s multiple comparison test. (TIF) [file pone.0039654.s007.tif]

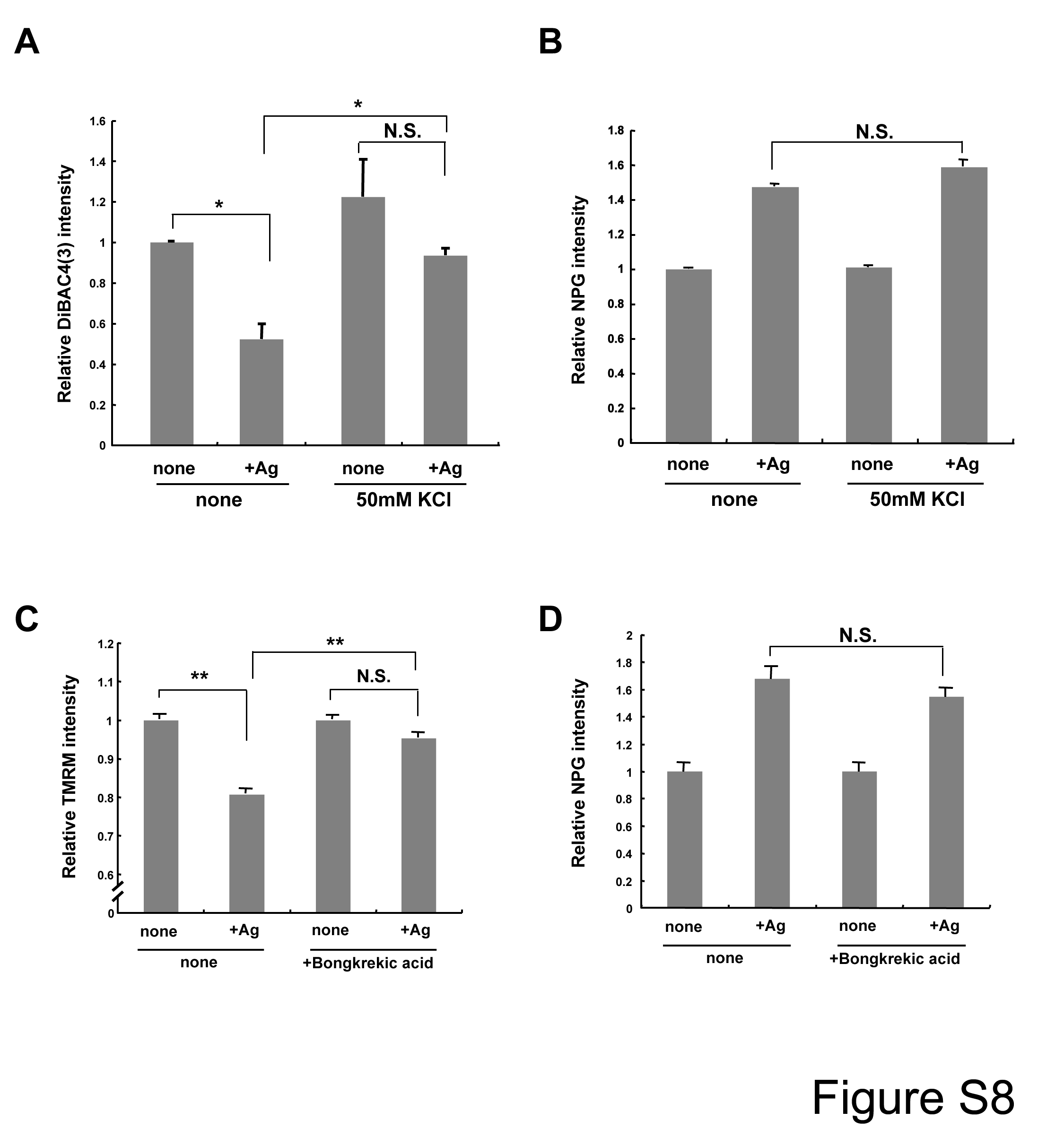

Supplement: Figure S8 — Effect of membrane potential changes on Zn-wave generation. (A) The plasma membrane potential was determined using a membrane potential-sensitive fluorescent dye DiBAC4(3) and flow cytometry. Data represent the mean fluorescent intensity of DiBAC4(3). (B) The effect of a high concentration of KCl on the FcεRI-mediated Zn wave was determined by examining the intracellular labile Zn level with or without 50 mM KCl treatment. Data represent the relative fluorescent intensity of Newport Green. (C) The intracellular membrane potential was determined with the cationic dye TMRM. BMMCs were treated with 20 µM Bongkrekic acid for 30 min before stimulation. The TMRM intensity of antigen-stimulated cells was determined by flow cytometry. Data represent the relative fluorescent intensity of TMRM. (D) The intracellular Zn level of BMMCs treated with or without 20 µM Bongkrekic acid upon antigen stimulation was determined by flow cytometry. Data represent the relative fluorescent intensity of Newport Green. N.S., not significant, *P<0.05, **P<0.01. Ag, Antigen; NPG, Newport Green. (TIF) [file pone.0039654.s008.tif]

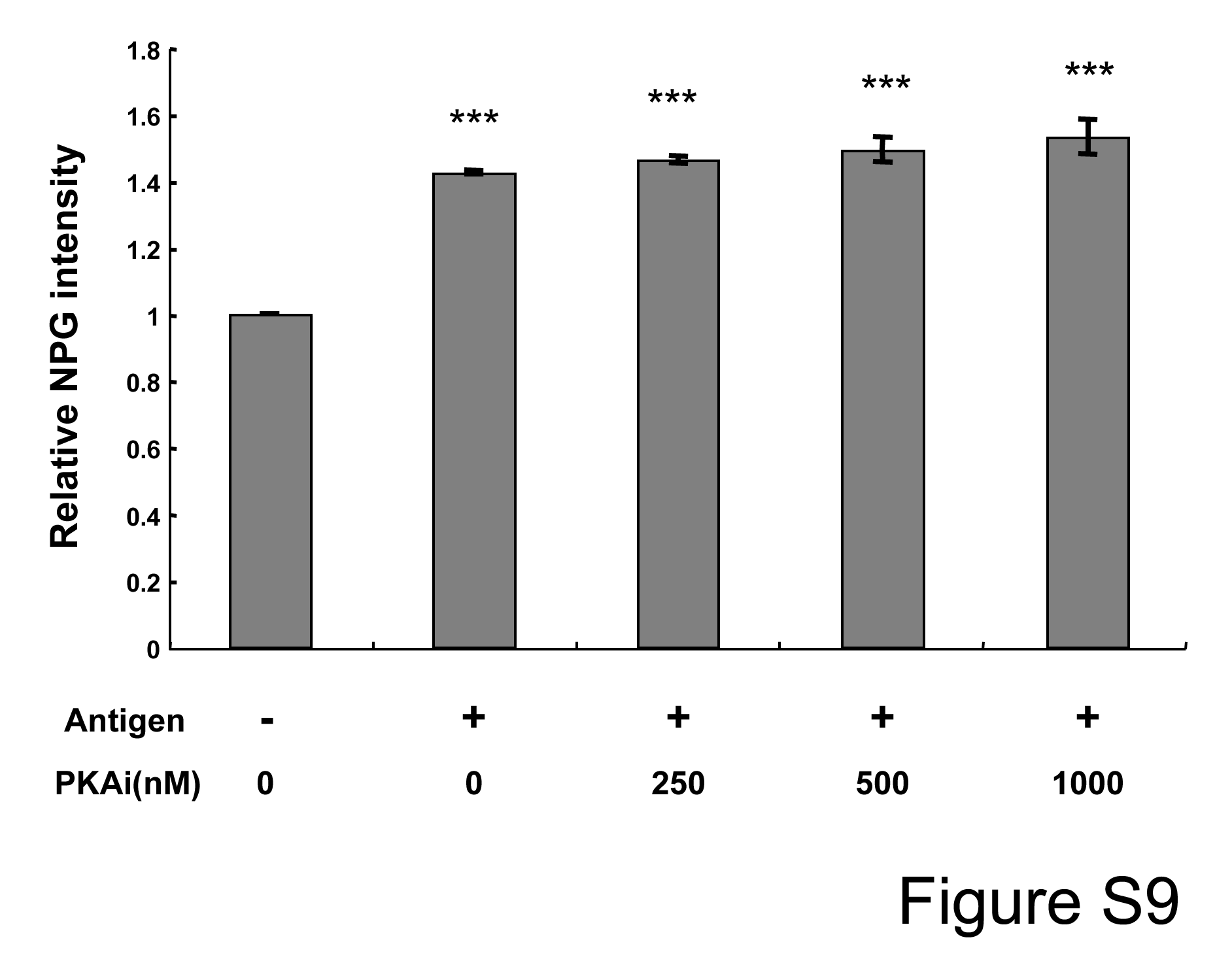

Supplement: Figure S9 — Effect of PKA-mediated signaling on the FcεRI-induced Zn wave. The FcεRI-mediated Zn wave was examined in BMMCs treated with a PKA inhibitor at the indicated concentration. The intracellular labile Zn level was determined by staining the BMMCs with Newport Green and analyzed by flow cytometry. Data represent the mean fluorescent intensity of Newport Green ± S.D. ***P<0.001 Student’s t-test, two-tailed. NPG, Newport Green. (TIF) [file pone.0039654.s009.tif]

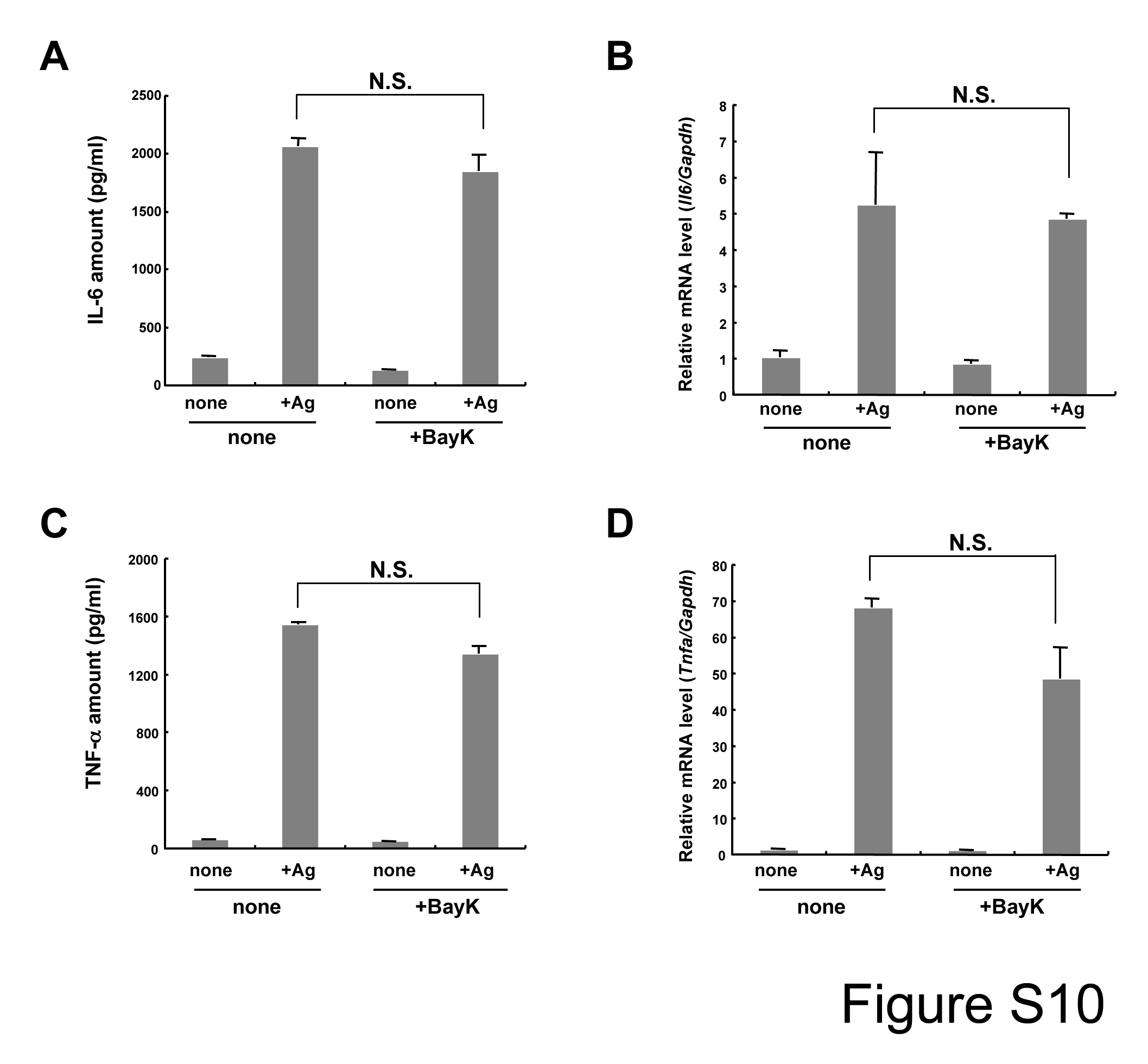

Supplement: Figure S10 — Effect of LTCC agonist-induced Zn elevation on cytokine production. The production of IL-6 and TNF-α upon antigen stimulation for 3 hours was measured by ELISA in BMMCs with or without pre-treatment with 20 µM (s)-(-)-BayK8644, an LTCC agonist in (A) and (C). FcεRI-mediated induction of Il6 and Tnfa transcription upon antigen stimulation in BMMCs with or without treatment with 20 µM (s)-(-)-BayK8644 was determined. The mRNA levels of Il6 and Tnfa were determined by semi-quantitative RT-PCR analysis in (s)-(-)-BayK8644-treated cells (B) and (D). N.S., not significant, Student’s t-test, two-tailed. Bay, (s)-(-)-BayK8644. (TIF) [file pone.0039654.s010.tif]
